# A Gait Pattern Generator for Closed-Loop Position Control of a Soft Walking Robot

**DOI:** 10.3389/frobt.2020.00087

**Published:** 2020-07-02

**Authors:** Lars Schiller, Arthur Seibel, Josef Schlattmann

**Affiliations:** ^1^Workgroup on System Technologies and Engineering Design Methodology, Hamburg University of Technology, Hamburg, Germany; ^2^Fraunhofer Research Institution for Additive Manufacturing Technologies IAPT, Hamburg, Germany

**Keywords:** mobile robotics, gait pattern generator, closed-loop position control, gecko-inspired soft robot, locomotion controller

## Abstract

This paper presents an approach to control the position of a gecko-inspired soft robot in Cartesian space. By formulating constraints under the assumption of constant curvature, the joint space of the robot is reduced in its dimension from nine to two. The remaining two generalized coordinates describe respectively the walking speed and the rotational speed of the robot and define the so-called velocity space. By means of simulations and experimental validation, the direct kinematics of the entire velocity space (mapping in Cartesian task space) is approximated by a bivariate polynomial. Based on this, an optimization problem is formulated that recursively generates the optimal references to reach a given target position in task space. Finally, we show in simulation and experiment that the robot can master arbitrary obstacle courses by making use of this gait pattern generator.

## 1. Introduction

Soft robotics is an emerging field in the robotics sciences and enjoys increasing attention in the scientific community (Bao et al., 2018). An important part of this field is mobile soft robotics, which allows locomotion in unknown and unstructured (Katzschmann et al., 2018) as well as potentially dangerous environments (Tolley et al., 2014). In order to navigate a robot through any environment, some sort of feedback is needed. As discussed in Santina et al. (2017), high gain feedback control results in good tracking performance, but imposes a reduction in the compliance of the controlled system. Therefore, it takes away the essential characteristic and greatest advantage of a soft robot—its softness (Rus and Tolley, 2015). When it comes to soft robots, usually the dynamics of inputs are indirectly coupled with the dynamics of outputs and the coupling is time-delayed (PneuNets: pressure—angle, SMA: heat—contraction, refer to Lee et al., 2013). In order to take this into account, a cascaded control architecture has been established (see, e.g., Marchese et al., 2014; Hofer and D'Andrea, 2018). In the case of pneumatically operated robots, the inner loop controls the pressure and the outer loop controls the pressure reference (see also **Figure 11B**). In order to preserve softness, the feedback gain of the outer control loop needs to be low. Most of the pressure reference should therefore be generated by a feed forward term (Santina et al., 2017). There is a trend to implement the feed forward term by using Iterative Learning Control (Bristow et al., 2006; see, e.g., Santina et al., 2017; Zhang and Polygerinos, 2018; Hofer et al., 2019). As shown in Santina et al. (2020), the typical soft properties of a soft robot can also be preserved with a model-based feed forward term when doing position control.

All the soft robots discussed so far are stationary. Thus, position control refers to the position of the end effector and not to the position of the entire robot. However, the same principles are also valid for mobile soft robots. Most mobile soft robots, such as in Shepherd et al. (2011), Godage et al. (2012), Tolley et al. (2014), Qin et al. (2019), and Schiller et al. (2019), are feed forward-controlled with predefined gait patterns. In order to enable such robots to move autonomously even in unknown terrain, a locomotion controller is needed that can generate any gait path. For solving this task, different methods have been employed, such as sine generators, central pattern generators (CPG), predefined trajectories, finite state machines, or heuristic control laws (Pratt et al., 2001). An example for sine generator-based locomotion control is presented in Horvat et al. (2015, 2017) for a salamander-like robot. The method enables to operate the robot by only two drive signals, i.e., forward and rotational speed. The main contribution here is the skilful synchronization of spine and legs motion, which is very robot-specific. In Ijspeert (2008), the suitability of central pattern generators, i.e., biologically inspired neural circuits capable of producing coordinated patterns for robot's locomotion, are discussed. It is concluded that CPGs are well-suited in general and especially for distributed implementations (e.g., for snake-like or reconfigurable robots). However, there is neither a sound design methodology to solve a specific locomotor problem nor a solid theoretical foundation. In order to implement CPGs in a meaningful way, the basic gait pattern must therefore be known from the outset, which again is robot-specific. An example for the automatic generation of optimal joint-trajectories is given in Bern et al. (2019). By using a forward shooting method and an FEM-based direct kinematics simulation, high-level goals, such as forward speed or direction of movement of various soft walking robots can be met. This method does not require a priori knowledge of a motion pattern, but can not be used online without restrictions (computation time, stability, .). However, it can be well used to find robot-specific gait patterns.

**Figure 1:**
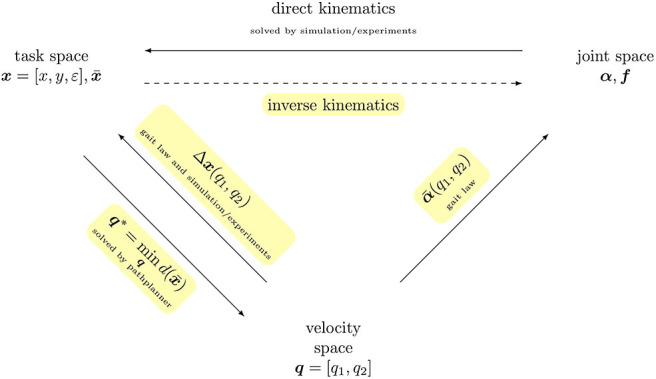
Overview of spaces: in order to approximate the inverse kinematics, the joint space of the robot is reduced by formulating constraints referred to as gait law $\bar{\alpha }$. The remaining two generalized coordinates ***q*** define the so-called velocity space. The direct kinematics of the entire velocity space (mapping in Cartesian task space) is approximated by a bivariate polynomial Δ***x***. By formulating an optimization problem $\min d\left(\bar{x}\right)$ that recursively generates the reference minimizing the distance to a given target position $\bar{x}$, the robot can be operated in task space.

Hence, for locomotion control of a robotic platform, a robot-specific motion strategy must be known. This paper analytically derives a robot-specific mapping of desired motion (forward and rotational speed) to joint coordinates for the gecko-inspired robot from Schiller et al. (2019), which is briefly described in section 2. The mapping function is referred to as “gait law” and is presented in section 3. In section 4, the direct kinematics of the robot are approximated by a polynomial by means of simulation and experiments to allow a fast evaluation. This is necessary to implement a control strategy in section 5 that maintains the softness of the robot and allows it to approach arbitrary references in the task space. The control strategy is referred to as Gait Pattern Generator. Figure 1 shows the systematic procedure of this paper. To summarize, the paper contributes in two ways: (i) it derives the robot-specific motion strategy for the gecko-inspired robot and (ii), for a given robot-specific motion strategy, it provides a method to control the robot's position. However, the underlying assumptions of the former can also be transferred to other soft robots, since the ability to adapt to the environment is exploited herein.

## 2. Robot and Experimental Setup

**Figure 2:**
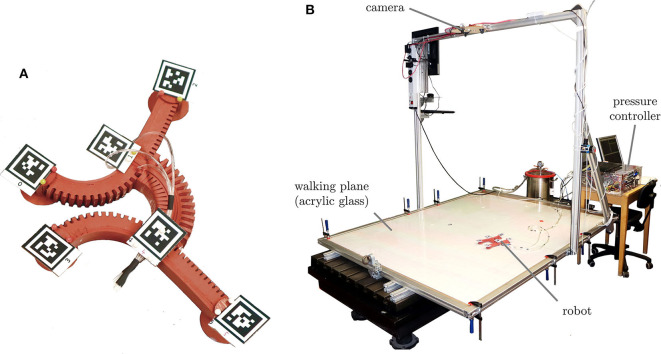
Experimental setup. **(A)** Prototype of the gecko-inspired soft robot with attached visual markers. **(B)** Test bench with embedded camera system for measuring the robot's position and evaluating the walking performance.

The soft robot this paper deals with has five limbs (four legs and a torso) and four feet that can be operated independently. Therefore, its joint space has nine dimensions: the five bending angles of the limbs **α** = [α_0_, α_1_, α_2_, α_3_, α_4_] and the four states of fixation actuators ***f*** = [*f*_0_, *f*_1_, *f*_2_, *f*_3_]. Since its locomotion is only possible within two dimensions, its description in task space needs only three coordinates: the *x* and *y* position of the robot ^*O*^*x* and its orientation ^*O*^ε, described in the global (Cartesian) coordinate system {*O*}. Thus, the task space has three dimensions. A photograph of the prototype of this robot is depicted in Figure 2A and Table 1 summarizes its specifications. In order to evaluate the performance of the robot, the test bench shown in Figure 2B was built with an embedded camera system. To measure the bending angles α, the robot orientation ε, and the robot position *x*, apriltags (Wang and Olson, 2016) were fixed on its body. For a more detailed description of the experimental setup, refer to the Supplementary Material.

**Table 1:** Specifications of the soft robot.

Total weight	150 g
Max. speed	6 cm/s
Body length	12 cm
Body span	17 cm
Average applied pressure	0.76 bar
Pull-off force of suction cups	47 N

## 3. Gait Law

The straight gait of the robot can be described by a single variable—the reference bending angle of the torso ${\bar{\alpha }}_{2}$. All other variables of the joint space can then be described as a function of ${\bar{\alpha }}_{2}$ by means of the gait law for the straight gait, which was derived in Seibel and Schiller (2018):

(1) $$\begin{array}{l} {\bar{\alpha }}_{\text{straight}}=\left[\begin{array}{c} \frac{\pi }{4}-\frac{{\bar{\alpha }}_{2}}{2}\\ \frac{\pi }{4}+\frac{{\bar{\alpha }}_{2}}{2}\\ {\bar{\alpha }}_{2}\\ \frac{\pi }{4}-\frac{{\bar{\alpha }}_{2}}{2}\\ \frac{\pi }{4}+\frac{{\bar{\alpha }}_{2}}{2} \end{array}\right],\;f=\left[\begin{array}{c} 0\text{ if }{\bar{\alpha }}_{2}<0\text{ else }1\\ 1\text{ if }{\bar{\alpha }}_{2}<0\text{ else }0\\ 1\text{ if }{\bar{\alpha }}_{2}<0\text{ else }0\\ 0\text{ if }{\bar{\alpha }}_{2}<0\text{ else }1 \end{array}\right]. \end{array}$$

For a constant cycle time, the torso's bending angle is the essential measure for the forward velocity. Therefore, *q*_1_ as the signal driving the forward velocity is introduced, and for straight gait, ${\bar{\alpha }}_{2}=q_{1}$ is set. In order to operate the robot with different velocities, the angle reference $\bar{\alpha }\left(q_{1}\right)$ for a given step size *q*_1_ is inverted after a certain time interval *t*_move_. Hence, it jumps from $\bar{\alpha }\left(q_{1}\right)$ to $\bar{\alpha }\left(-q_{1}\right)$. The corresponding fixation reference *f* must also be inverted.

### 3.1. Derivation for General Case

The above gait law can only generate gait patterns for straight motion. It is based on the idea that the orientations of the feet always remain constant. Now, we will loosen this restriction and demand only constant orientations for the fixed feet, while the unfixed feet are allowed to rotate. This implies two cases to be considered:

1. What should be the rule for a fixed foot so that its orientation remains constant regardless of the rotation of the body?
2. What should be the rule for a free foot so that its change of orientation matches that of the body and enables a suitable initial pose for the next cycle?

For both cases, the rules are based on the change of orientations of the feet. The orientations of feet $\varphi ={\left[\varphi_{0}\;\varphi_{2}\;\varphi_{3}\;\varphi_{5}\right]}^{⊤}$ described in the global coordinate system—and consequently their change during the change of pose—can be calculated assuming constant curvature as follows:

(2) $$\begin{array}{r} \varphi \left(\alpha ,\varepsilon \right)=\left[\begin{array}{c} \varepsilon -\frac{1}{2}\alpha_{2}-\alpha_{0}\\ \varepsilon -\frac{1}{2}\alpha_{2}+\alpha_{1}\\ \varepsilon +\frac{1}{2}\alpha_{2}+\alpha_{3}+\pi \\ \varepsilon +\frac{1}{2}\alpha_{2}-\alpha_{4}+\pi \end{array}\right]\;. \end{array}$$

**Figure 3:**
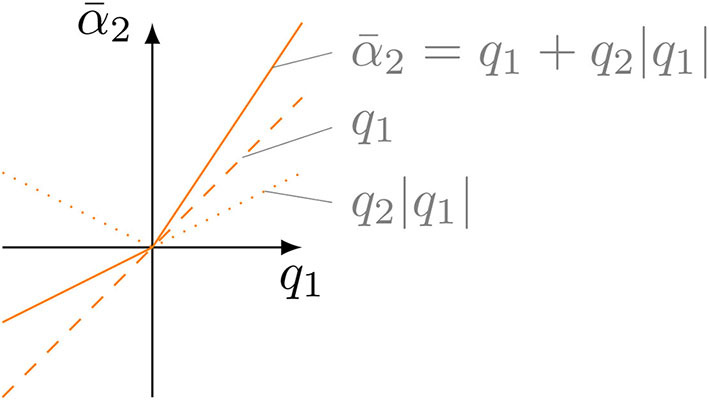
Illustration of how the steering *q*_2_ influences the reference angle of the torso ${\bar{\alpha }}_{2}$.

**Figure 4:**
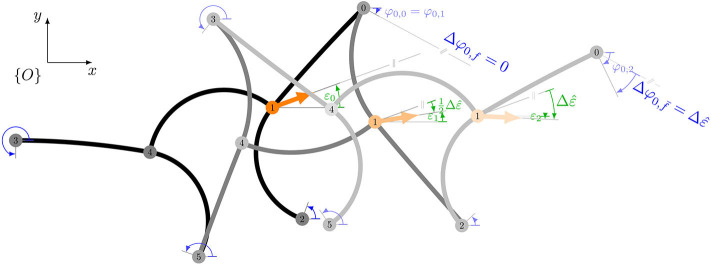
Problem statement: which bending angles must be applied in order to turn the robot while keeping the orientation of its fixed feet? During the first change of pose, the orientation of the front left foot (φ_0_) and the rear right foot (φ_5_) should be kept constant. During the second change of pose, the front right foot (φ_2_) and the rear left foot (φ_3_) should not rotate.

Since the feet's orientations depend on the robot's orientation ε and the bending angles **α**, a description for the latter two is required. First, it will be discussed how to describe and how to change the walking direction of the robot ε, i.e., its orientation. From Schiller et al. (2020), it is known that the asymmetrical actuation of the torso leads to a rotation of the body. In order to describe an asymmetrical actuation, the steering factor *q*_2_ is introduced. The reference angle for the torso ${\bar{\alpha }}_{2}$ is then described as follows:

(3) $$\begin{array}{l} {\bar{\alpha }}_{2}=q_{1}+|q_{1}|q_{2}, \end{array}$$

where *q*_2_ ∈ [−0.5, 0.5] is dimensionless and shifts the reference angle of the torso ${\bar{\alpha }}_{2}$ in the direction of *q*_2_ (compare Figure 3). In this way, the left side of the torso is actuated by |*q*_1_|*q*_2_ more in the first half of a cycle and the right side by the same amount less in the second half of the cycle. It should be noted that Equation (3) describes only one possible model for asymmetric actuation. Several models have been tested and this one has been established. Clearly, the change of orientation per cycle Δε is related to the steering factor *q*_2_ and the step length *q*_1_. Simulation and experiment show that, for asymmetric actuation with positive *q*_2_, a negative change of orientation occurs, and vice versa. The change in orientation per cycle is therefore negatively proportional to the steering factor and the step length:

$$\begin{array}{l} \Delta \varepsilon \sim -|\Delta q_{1}|q_{2}\;, \end{array}$$

where |Δ*q*_1_| is the amount of change in torso actuation from initial pose to subsequent pose. This results in a model for orientation change per cycle $\Delta \hat{\varepsilon }$ of the body:

(4) $$\begin{array}{l} \frac{1}{2}\Delta \hat{\varepsilon }=-{\tilde{c}}_{1}|\Delta q_{1}|q_{2}\;, \end{array}$$

where the robot-specific constant ${\tilde{c}}_{1}$ describes the ability of the robot to rotate. Here, it is assumed that the robot rotates consistently within the cycle. Therefore, the change in orientation after a pose change (half cycle) is exactly half as much as after the entire cycle; compare to Figure 4 where $\frac{1}{2}\Delta \varepsilon =\varepsilon_{1}-\varepsilon_{0}=\varepsilon_{2}-\varepsilon_{1}$.

The second parameter for calculating the feet's orientations Equation (2) is the bending angles of the legs. Hence, a specification for the legs is needed. The structure of the straight gait law from Equation (1) was adopted for this purpose, whereby the reference angles of the legs are extended with a yet unknown term *g*(*q*_1_, *q*_2_). In the following, the procedure is shown for the front left leg only (α_0_). However, it can be transferred to all other legs. With this extension, the reference angle for the front left leg results in:

(5) $$\begin{array}{l} {\bar{\alpha }}_{0}=\frac{\pi }{4}-\frac{{\bar{\alpha }}_{2}}{2}+g\left(q_{1},q_{2}\right)\;. \end{array}$$

Now, the change of foot orientation when changing the pose Δφ can be derived from Equations (2)–(5) by treating the references of the bending angles as the actual bending angles and assuming the body rotates according to the model from Equation (4):

(6) $$\begin{array}{l} \begin{array}{l} \Delta \varphi_{0}=\;\varphi_{0,1}-\varphi_{0,0}\\ \;=\;\left(\varepsilon_{1}-\frac{{\bar{\alpha }}_{2,1}}{2}-{\bar{\alpha }}_{0,1}\right)-\left(\varepsilon_{0}-\frac{{\bar{\alpha }}_{2,1}}{2}-{\bar{\alpha }}_{0,0}\right)\\ \;=\;\left(\varepsilon_{1}-\frac{\pi }{4}-g\left(q_{1,1},q_{2}\right)\right)-\left(\varepsilon_{0}-\frac{\pi }{4}-g\left(q_{1,0},q_{2}\right)\right)\\ \;=\;\left(\varepsilon_{1}-\varepsilon_{0}\right)-g\left(q_{1,1},q_{2}\right)-g\left(q_{1,0},q_{2}\right)\\ \;=\;\frac{1}{2}\Delta \hat{\varepsilon }-\Delta g\left(q_{1},q_{2}\right) \end{array}\;, \end{array}$$

where *q*_1, 0_ describes the step length of the initial pose and *q*_1, 1_ that of the subsequent pose. When changing poses, the robot always jumps from $\bar{\alpha }\left(q_{1},\cdot \right)$ to $\bar{\alpha }\left(-q_{1},\cdot \right)$. Therefore, *q*_1, 1_ = −*q*_1, 0_ and *g*(*q*_1, 1_, *q*_2_) − *g*(*q*_1, 0_, *q*_2_) can be combined to Δ*g*(*q*_1_, *q*_2_). Furthermore, it is assumed that the steering factor *q*_2_ remains unchanged when changing poses. Next, a specification for the additional term *g*(*q*_1_, *q*_2_) is derived for the two cases under consideration (fixed and unfixed leg).

#### 3.1.1. Fixed Leg

Figure 4 shows one cycle of trotting gait. Within the transition from the initial pose (black) to the middle pose (gray), the front left foot is fixed and thus its orientation should remain constant. The bending angle must be determined in such a way that the foot's orientation is kept constant, i.e., independent of *q*_1_ or *q*_2_:

(7) $$\begin{array}{l} \Delta \varphi_{0,f}=\frac{1}{2}\Delta \hat{\varepsilon }-\Delta g_{f}\left(q_{1},q_{2}\right)=0\;\forall q_{1},q_{2}\;, \end{array}$$

where the index *f* denotes a fixed foot/leg. This means that the robot can change from any pose described by the general gait law to a subsequent pose without changing the orientation of its fixed feet, with the limitation that the steering factor *q*_2_ remains constant with this change. From Equations (7) and (4), the additional term for the fixed leg results in:

(8) $$\begin{array}{l} \Delta g_{f}\left(q_{1},q_{2}\right)=\frac{1}{2}\Delta \hat{\varepsilon }=-{\tilde{c}}_{1}|\Delta q_{1}|q_{2}\;. \end{array}$$

Since the sign of *q*_1_ is always swapped when changing poses, the change of the torso actuation always results in |Δ*q*_1_| = 2|*q*_1_|, and thus, the additional term becomes (with $c_{1}=-4{\tilde{c}}_{1}$):

(9) $$\begin{array}{l} g_{f}\left(q_{1},q_{2}\right)=c_{1}|q_{1}|q_{2}\;. \end{array}$$

Inserted in Equation (5), the reference for a fixed leg results in:

(10) $$\begin{array}{l} \alpha_{0,f}=\frac{\pi }{4}-\frac{q_{1}}{2}-\frac{1}{2}|q_{1}|q_{2}+c_{1}|q_{1}|q_{2}\;. \end{array}$$

#### 3.1.2. Free Leg

As the foot was previously fixed, the rotation of the body must affect its orientation in the non-fixed phase. The free foot should therefore rotate in the unfixed phase exactly as much as the body does during the entire cycle. This is illustrated in Figure 4 where the change in orientation of the front left foot between the final pose (lightgray) and the middle pose (gray) matches exactly the rotation of the body $\Delta \hat{\varepsilon }$. With the model for the change of foot orientation from Equation (6), it must hold:

(11) $$\begin{array}{l} \Delta \varphi_{0,\bar{f}}=\frac{1}{2}\Delta \hat{\varepsilon }-\Delta g_{\bar{f}}\left(q_{1},q_{2}\right)=\Delta \hat{\varepsilon }\;\forall q_{1},q_{2}\;, \end{array}$$

where $\bar{f}$ indicates an unfixed foot. Clearly, this only applies if the same additional term is added again, but with swapped sign:

(12) $$\begin{array}{l} g_{\bar{f}}\left(q_{1},q_{2}\right)=-g_{f}\left(q_{1},q_{2}\right)=-c_{1}|q_{1}|q_{2}\;. \end{array}$$

According to Equation (5), the reference for a free leg results in:

(13) $$\begin{array}{l} \alpha_{0,\bar{f}}=\frac{\pi }{4}-\frac{q_{1}}{2}-\frac{1}{2}|q_{1}|q_{2}-c_{1}|q_{1}|q_{2}\;. \end{array}$$

If a foot is fixed, we add the term *g*(*q*_1_, *q*_2_) = *c*_1_|*q*_1_|*q*_2_ to the reference angle of the corresponding leg. If the leg is free, the additional term *g* is subtracted. Whether a leg is fixed or not is determined by the sign of the torso reference (see Equation 1): *q*_1_ positive → foot fixed, *q*_1_ negative → foot free. Thus, the distinction between free and fixed leg can be avoided by dropping the amount operation of *q*_1_ in the additional term *g*. The sign of *q*_1_ then automatically controls the corrective direction of the additional term *g*. This procedure can be performed for all legs and results in the general gait law, which is formally described as follows:

(14) $$\begin{array}{l} \bar{\alpha }=\left[\begin{array}{c} \frac{\pi }{4}-\frac{q_{1}}{2}-\frac{1}{2}|q_{1}|q_{2}+c_{1}q_{1}q_{2}\\ \frac{\pi }{4}+\frac{q_{1}}{2}+\frac{1}{2}|q_{1}|q_{2}+c_{1}q_{1}q_{2}\\ q_{1}+|q_{1}|q_{2}\\ \frac{\pi }{4}-\frac{q_{1}}{2}-\frac{1}{2}|q_{1}|q_{2}+c_{1}q_{1}q_{2}\\ \frac{\pi }{4}+\frac{q_{1}}{2}+\frac{1}{2}|q_{1}|q_{2}+c_{1}q_{1}q_{2} \end{array}\right],\;f=\left(1\right)\;. \end{array}$$

**Figure 5:**
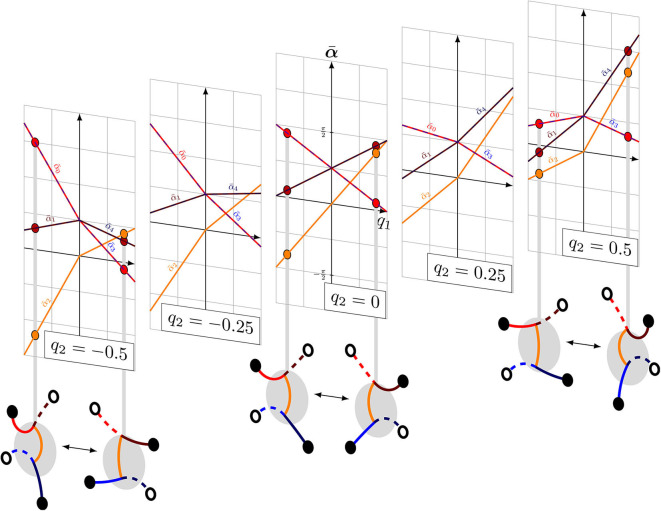
Visualization of the velocity space defined the by general gait law from Equation (14) for *q*_2_ ∈ {−0.5, −0.25, 0, 0.25, 0.5}.

The value of additional leg bending *c*_1_ is to be determined via simulations or experiments. This is demonstrated in the Supplementary Material and results in *c*_1_ = 1. The visualization of this law is shown in Figure 5. Note that the middle layer shows the special case for straight motion from Equation (1). By introducing the index *k* specifying the extreme poses, references for a gait can be generated recursively by

(15) $$\begin{array}{l} {\bar{\alpha }}_{k}=\bar{\alpha }\left(-q_{1,k-1},q_{2}\right),f_{k}=\neg f_{k-1}, \end{array}$$

where ¬***f*** is the logical negation of ***f***.

The gait law generates reference angles for the robot, depending on step length (forward velocity) *q*_1_ and steering factor (rotational velocity) *q*_2_. These two generalized coordinates define the so-called velocity space of trotting gaits, since each pair (*q*_1_, *q*_2_) describes another trotting gait. If *q*_1_ and *q*_2_ remain constant during gait, theoretically, the orientation of the fixed feet does not change. However, the derivation of this law did not examine whether the fixed feet also remain in position when switching poses. Also, the robot should have the ability to change its gait over time and should not always run the same circle with the same velocity. Therefore, *q*_1_ and *q*_2_ must vary. The next section examines whether this law provides useful references, despite neglecting the feet positions.

### 3.2. Experimental Validation

**Figure 6:**
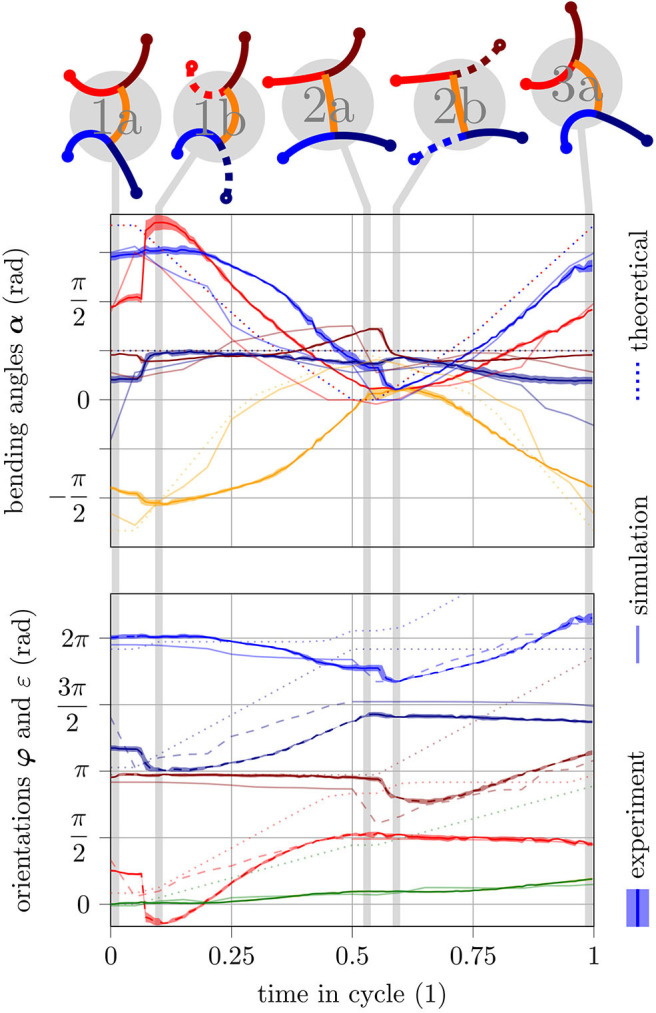
Simulation and experiment of one gait cycle for *q* = [80° − 0.5] and *c*_1_ = 1. Theoretical values (according to the gait law) are illustrated with light dotted lines. Simulated values are illustrated with light solid lines. Experimental values are illustrated as solid lines together with an area indicating the standard deviation. In the orientation plot and the poses shown above, lines representing unfixed feet/legs are illustrated as dashed lines. The switch of fixation happens at half cycle time. Color code as follows: front left leg (red), front right leg (dark red), torso (orange), rear left leg (blue), rear right leg (dark blue), robot orientation (green).

Within an experiment, it shall be analyzed whether the orientations of fixed feet actually remain constant during a cycle or ignoring the feet positions leads to significant discrepancies. The gait was slowed down (*t*_move_ = 10 s) as highly dynamic changes smear the camera images and the tags can no longer be detected by image processing. Figure 6 shows an exemplary cycle of a gait for $q_{1}=80^{\circ }$ and *q*_2_ = −0.5. The figure shows the mean values and standard deviations of five experiments in total. For the detailed processing steps in the evaluation, refer to the Supplementary Material. The upper graph shows the progression of the bending angles α and the lower graph shows the progression of the orientations φ and ε during a cycle. Initially, all feet are fixed (pose 1a). The bending angle of the front left (red line) and the rear right leg (dark blue line) differs significantly from the reference at this point in time because the robot is forced into this pose by the fixation of its feet. After about five percent of the cycle time, the front left and rear right foot are released (pose 1b). At this point, a jump in the bending angle of the two corresponding legs can be observed—the angles jump to their reference. The same effect can be observed when changing the feet fixation in the middle of the cycle (pose 2a → 2b). From this observation, it can be deduced that the robot cannot match the reference generated by the gait law because the closed kinematic chain of its parallel structure prevents it from adopting the specified bending angles. The φ graph shows that the orientations of the fixed feet remain nearly constant as assumed when deriving the gait law. An exception is the rear left foot (blue line): its orientation changes significantly during the fixed phase. As already seen in Schiller et al. (2020), the suction cups of the robot have a certain margin of rotation. This must now be utilized; otherwise, the feet would have to move (which is not possible because of the fixation). In summary, it can be concluded from the experiment in Figure 6 that the gait law provides references which cannot be fully realized due to the closed kinematic chain, but nevertheless lead to the desired behavior.

## 4. Approximating the Direct Kinematics

The next step is to determine how the robot behaves in the task space for each point (*q*_1_, *q*_2_) in the velocity space—that is, how it moves per cycle and by how much it rotates. Thus, the bivariate polynomial Δ*x*(*q*_1_, *q*_2_) is searched for which approximates the transformation of the velocity space into the task space (compare Figure 1). The form of the polynomial is defined as follows:

(16) $$\begin{array}{l} \Delta x\left(q_{1},q_{2}\right)=\left[\begin{array}{c} \Delta \varepsilon \\ \Delta x\\ \Delta y \end{array}\right],\Delta \varepsilon ,\Delta x,\Delta y:=\underset{i,j}{\sum }a_{i,j}q_{1}^{i}q_{2}^{j}\;. \end{array}$$

In order to identify the coefficients, the velocity space is gridded and for each set of values the motion of the robot is measured. This can either be done experimentally or the simulation model is used and the movement is simulated. The result of both approaches depends on the way they are implemented. Therefore, the influencing factors must be identified and their value must be meaningfully determined. Table 2 summarizes the conditions under which the following experiments or simulation were carried out. A detailed discussion of the experimental conditions can be found in the Supplementary Material.

**Table 2:** Influencing factors on simulation and experiment.

Initial pose	$\alpha_{0}=\bar{\alpha }\left(q_{1},q_{2}\right)$, *f*_0_ = [1, 0, 0, 1]
Number of cycles	min. *n*_cyc_ = 2, drop initial cycle
Dimensions of robot	ℓ_leg_ = 9.1 cm, ℓ_torso_ = 10.3 cm
Weighting parameters (only simulation)	*f*_*l*_, *f*_*o*_, *f*_*a*_ = 89, 10, 5.9
Model order of *Δx*(*q*_1_, *q*_2_)	order = 2

**Figure 7:**
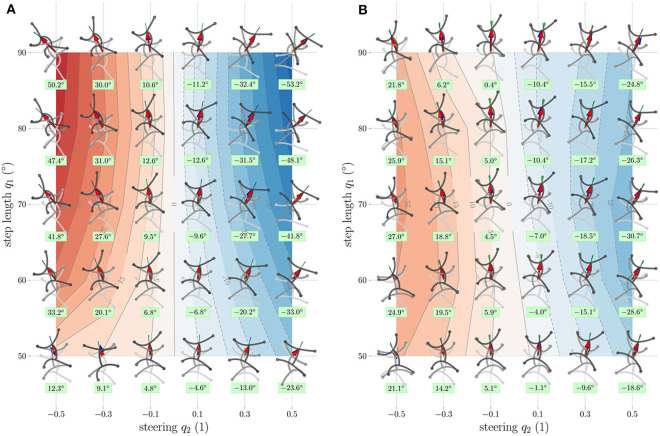
Resulting experimental gaits according to the gait law in Equation (14) for a variation of step length and steering factor. The rows each have a constant step length *q*_1_ and the columns a constant steering factor *q*_2_. Each frame shows the resulting motion of one cycle with the pattern corresponding to (*q*_1_, *q*_2_). Below each frame, the rotation per cycle in degrees Δε is stated. The heat map in the background shows the polynomial fit of Δε. The bold red vector pointing from the initial position of each individual gait to its end position is called [Δ*x*Δ*y*]^⊤^. **(A)** Simulation (89, 10, 5.9) and **(B)** experiment.

**Figure 8:**
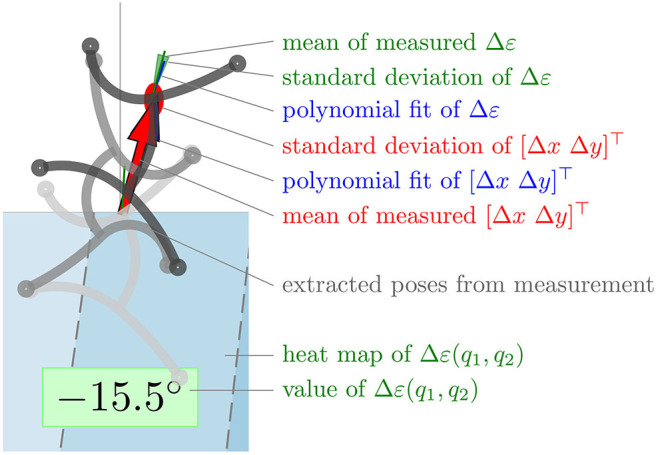
Detailed view of resulting experimental gait according to the gait law for $\left(q_{1},q_{2}\right)=\left(90^{\circ },0.3\right)$.

Figure 7 shows the results of simulation (Figure 7A) and experiment (Figure 7B). In both cases, the velocity space was gridded with *q*_1_ ∈ {50, 60, ⋯ , 90} and *q*_2_ ∈ {−0.5, −0.3, ⋯0.5} and a measurement was performed for each grid point. A simplified representation of the extreme poses of the resulting gait illustrates the movement. The tip of the torso of the initial pose is always at the position (*q*_1_, *q*_2_) and the orientation of the robot faces upwards. The resulting translation [Δ*x*Δ*y*]^⊤^ per cycle is indicated by a red arrow. Besides, the orientation of the robot after a cycle is represented by a green line. The heat map in the background shows the resulting rotation Δε(*q*_1_, *q*_2_) per cycle. The numerical value of this function is noted in a green box below the individual measurements. In the figure of the experiment (Figure 7B), the standard deviation of the translation is shown as a red ellipse with the corresponding semi axes. The standard deviation of the rotation is visualized as a light green triangle with an opening angle of 2std(δε). The blue arrow shows the polynomial fit of the translation and the blue line the polynomial fit of the rotation at the corresponding grid point. A detailed view of a single experiment is shown in Figure 8.

In contrast to the experiment in section 3.2, in Figure 7, a clear deviation between simulated and experimental results can be observed. The resulting rotation and the shift in transverse direction are noticeably higher for all grid points. The simulation model does not reproduce friction effects or external disturbances, such as the influence of the supply tubes. In the previous experiment (from section 3.2), these effects played a subordinate role because of the reduced speed and the relatively short distance traveled. This experiment was executed at full speed (*t*_move_ = 1 s); thus, friction has a significantly larger influence. Furthermore, we can observe that the experiment is not symmetrical, meaning that swapping the sign of *q*_2_ does not yield to mirrored behavior [Δ***x***(*q*_1_, *q*_2_)≁−Δ***x***(*q*_1_, −*q*_2_)]. This can be attributed to manufacturing inaccuracies of the robot and an optimizable pressure-bending angle calibration. The calibration procedure and associated difficulties are also discussed in the Supplementary Material. A final observation is that, in the experiment, the resulting rotation decreases for a large step length *q*_1_. This is different to the simulation, where the resulting rotation increases steadily with increasing step length. For large values of *q*_1_ and *q*_2_, the gait law prescribes relatively large reference angles. If these are out of range of calibration of the respective actuator, the reference pressure is saturated to prevent damage to the robot. Exactly this effect occurs in the upper part ($q_{1}\geq 80^{\circ }$) of Figure 7B. Therefore, the poses here deviate much more from their simulated counterparts in Figure 7A than in the lower part of the figure ($q_{1}<80^{\circ }$). Apart from the “over-simulation” and the missing saturation effect, the simulation reproduces the behavior very well. It can be seen as the behavior of a robot that has been perfectly manufactured and calibrated, consisting of actuators as robust as saturation is no longer necessary, whose feet have the optimum torsional stiffness, and where all friction effects have been reduced to a minimum. For the implementation of the Gait Pattern Generator, however, the actual interest focuses on the polynomial fit of the motion. In most cases, the second-order fit shown in blue matches the measurement or is at least within the standard deviation. The coefficients for the polynomial Δ***x***(*q*_1_, *q*_2_) for Equation (16) are listed in Table 3.

**Table 3:** Coefficients of the bivariate polynomial fit of the motion per cycle Δ*x* for the experiment.

***f*(*q*_1_, *q*_2_)** **= **	***a*_0, 0_**	***a***_1, 0_·*q*_1_	***a*_0, 1_·*q*_2_**	**$a_{1,0}.\;q_{1}^{2}$**	**$a_{2,0}.\;q_{2}^{2}$**	***a***_1, 1_·*q*_1_*q***_2_**
Δε (°)	5.4154	−0.0457	−44.1944	−0.0006	0.778	−0.0832
Δ*x* (cm)	0.1106	0.2225	11.4146	−0.0008	−17.5133	−0.1213
Δ*y* (cm)	1.9498	−0.0682	−3.6997	0.0004	−0.0333	−0.0580

## 5. Gait Pattern Generator

The last step to control the robot's position is the calculation of the optimal tuple q^*^ to move from the current position ***x*** closer to a given target position $\bar{x}$ (compare Figure 1).

### 5.1. Derivation

As derived in section 4, the robot turns around Δε and moves by [Δ*x*Δ*y*]^⊤^ with each cycle. Therefore, the position of the (*n*+1)th pose given in the coordinate system of the *n*th pose can be described by

(17) $$\begin{array}{l} R^{\left(n\right)}x_{\left(n+1\right)}=\left[\begin{array}{c} \Delta x(q_{1},q_{2})\\ \Delta y(q_{1},q_{2}) \end{array}\right]\;, \end{array}$$

where the index *n* starts from 0 indicating the initial pose and accordingly the subsequent poses. If step length *q*_1_ and steering factor *q*_2_ do not change during gait (***q*** = const.), the translation and rotation per cycle will remain the same. Let us assume that it would be possible to reach the target position in a finite number of cycles without changing the gait. Accordingly, the vector ${\;}^{R^{\left(n\right)}}{\bar{x}}_{\left(n\right)}$ pointing from the *n*th pose to the target, can be described in the coordinate system of the *n*th pose as a function of the target vector of the (*n* − 1)th pose:

(18) x¯R(n) (n)=R(−Δε)(R(n−1)x¯(n−1)−R(n−1)x(n)),

where *R* ∈ ℝ^2 × 2^ is the rotation matrix. Since for multiple rotations around the same axis ***R***^*k*^(*x*) = ***R***(*kx*) applies, this can be formulated explicitly:

(19) $$\begin{array}{l} {\;}^{R^{\left(0\right)}}{\bar{x}}_{\left(n\right)}=R{\left(-n\Delta \varepsilon \right)}^{R^{\left(0\right)}}{\bar{x}}_{0}-\overset{n}{\underset{i=0}{\sum }}R\left(-i\Delta \varepsilon \right)\left[\begin{array}{c} \Delta x\\ \Delta y \end{array}\right]\;. \end{array}$$

**Figure 9:**
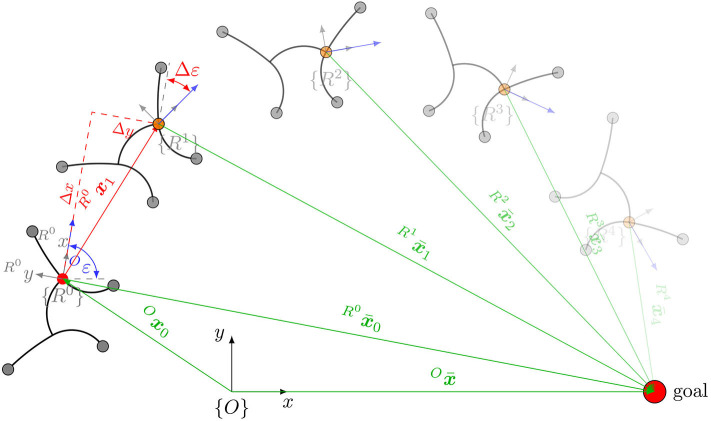
Visualization of Equations (17)–(22). By using the approximation of the direct kinematics Δ*x*, the approximated position in Cartesian space after *n* cycles can be easily calculated. In the figure, the opacity decreases with increasing cycle number.

Figure 9 visualizes these formulas, whereby the opacity of poses that lie further in the future decreases. Now, the distance *d*_*n*_ to the target position $\bar{x}$ after *n* cycles of trotting with the pattern corresponding to the gait law $\bar{\alpha }\left(q_{1},q_{2}\right)$ can be calculated with

(20) $$\begin{array}{l} d_{n}{(}^{R^{\left(0\right)}}{\bar{x}}_{0},\Delta \;x)=|R^{\left(0\right)}{\bar{x}}_{\left(n\right)}{|}_{2}\;. \end{array}$$

For a given target, the optimal tuple for *n* cycles can then be calculated as the minimum of the distance function

(21) $$\begin{array}{l} q_{1}^{*},q_{2}^{*}=\underset{q_{1},q_{2}\in Q}{\min}d_{n}^{2}\left({\;}^{R^{\left(0\right)}}{\bar{x}}_{0},\Delta \;x\right)\;, \end{array}$$

where Q describes the set of feasible values for *q*_1_ and *q*_2_, respectively. Note that the vector ${\;}^{R^{\left(0\right)}}{\bar{x}}_{0}$ describes the target position in the coordinate system of the initial pose. In the test bed with an external camera measurement system, this vector must be calculated from the measurements of the current pose ^*O*^*x* and the target position ${\;}^{O}\bar{x}$:

(22) $$\begin{array}{l} {\;}^{R^{\left(0\right)}}{\bar{x}}_{0}=\;R({-}^{O}\varepsilon )\left({\;}^{O}\bar{x}{-}^{O}x\right)\;. \end{array}$$

However, the target measurement could also happen with a camera directly mounted on the robot without having to reformulate the equations, as the Gait Pattern Generator demands the target position in the robot coordinate system.

**Figure 10:**
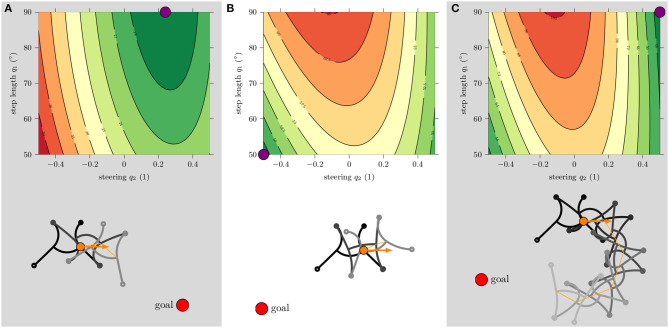
Evaluation of the distance function *d*_*n*_ for different target positions $\bar{x}$ and planning horizons *n*. The lowest values are represented by green and the highest values by red color. The lower image always shows the resulting simulated gait for *n* cycles, corresponding to the minimum distance (marked by a purple circle). Simulations were initialized with: ^*O*^*x* = (0, 0), ^*O*^ε = 0°, α_0_ = [90 0−90 90 0], *f*_0_ = [1 0 0 1]. **(A)** Planning horizon *n* = 1 for target at $\bar{x}=\left(35,-20\right)$, **(B)** *n* = 1 for target at $\bar{x}=\left(-35,-20\right)$, and **(C)** *n* = 4 for target at $\bar{x}=\left(-35,-20\right)$.

Figure 10 shows a visualization of the distance function *d*_*n*_ for different target points and the patterns corresponding to its minimum. In Figure 10A, the target is located slanted right in front of the robot and a planning horizon of *n* = 1 is considered. The minimum of the distance function is at full step length $q_{1}=90^{\circ }$ and a medium steering factor *q*_2_ = 0.3. The resulting reference allows the robot to move precisely to the front right. In Figure 10B, the target is located behind the robot. With a planning horizon of *n* = 1, the minimum distance results in the smallest allowed step length and steering. However, this solution does not bring the robot closer to the target, but it is the solution that minimizes the increase in distance. There is simply no gait pattern that can bring the robot closer to the target within only one cycle. For this reason, the planning horizon in Figure 10C was increased to *n* = 4. The minimum of *d*_*n* = 4_ is now at maximum step length and maximum steering factor for the same target position. The resulting reference leads to the desired behavior: the tightest possible right turn.

### 5.2. Implementation

**Figure 11:**
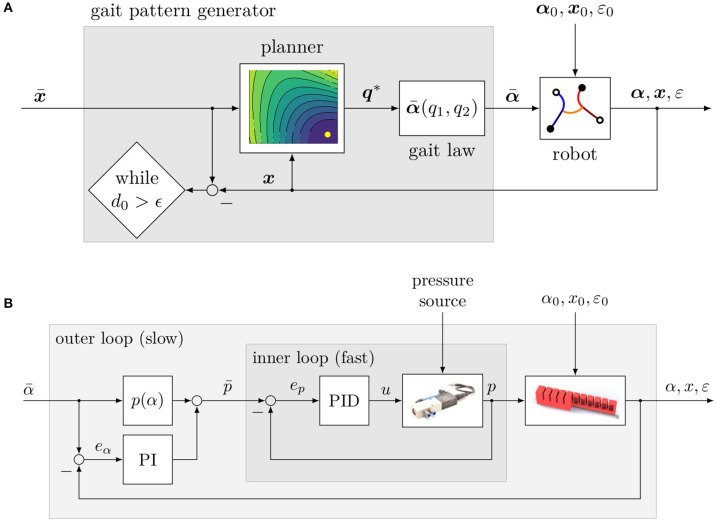
Control architecture of the Gait Pattern Generator. **(A)** For a given target position $\bar{x}$ and the position of the robot *x*, the optimal step length *q*_1_ and steering factor *q*_2_ are calculated and then mapped into reference bending angles $\bar{\alpha }\left(\cdot \right)$ by the gait law, which are then fed into the robotic system. In **(B)**, the block diagram of a single actuator is shown. The reference bending angle $\bar{\alpha }$ is mapped by a calibration function *p*(α) into a reference pressure $\bar{p}$ (feed forward term), which in turn is corrected by a saturated PI controller (feedback term). The reference pressure is then fed into the inner loop, where a PID controller generates the control input *u* for a proportional valve, which causes the pressure *p* to be applied to the actuator.

As seen in the previous section, the distance to the target cannot always be reduced in just one cycle. The simplest strategy to solve this problem is to incrementally increase the planning horizon as long as the minimum possible distance to the target within the next *n* cycles *d*_*n*, min_ is larger than the current distance *d*_0_. Furthermore, a strategy for transitioning between different gait patterns is required. So far, all simulations and experiments have only studied the motion of consistent gaits (***q*** = const.). However, the pattern generator should be able to dynamically change both step length and steering factor. The easiest way to make this possible is to assume that the robot is able to switch between any gait pattern, which means to allow all possible references regardless of the current pose. Here, it is questionable whether the output q^*^ actually minimizes the distance to the target or whether another solution might be more suitable, since the calculation in most cases will be based on a different initial pose. Thus, it can be assumed that a different q^*^ would be calculated when considering the current pose of the robot. But by feeding back the current position after each step and a recalculation of the reference, reaching the target position can still be ensured. Algorithm 1 implements exactly this strategy. Figure 11A shows the procedure as a block diagram. The sampling rate of this control loop depends on the length of half a cycle and is slightly less than 1 Hz. The Gait Pattern Generator is paused as soon as the actual distance to the target is less than a defined value ϵ = 5 cm. For better comprehension, Figure 11B shows the low-level control architecture of the robotic system for a single actuator. Note that the simulation model mimics the coupled behavior of six of these blocks.

**Algorithm 1:** **Gait Pattern Generator**.

1: **procedure** Generate reference (${\bar{x}}_{k}$)
2: *n* ← 1 ⊳ start with planning horizon of 1 cycle
3: $q_{k}^{*},\;d_{n,\text{min}}\leftarrow \underset{q}{\min}d_{n}\left(\bar{x},\Delta x\left(q\right)\right)$ ⊳ minimal distance to goal after 1 cycle
4: **while** *d*_0_ > *d*_*n*, min_ **do** ⊳ stop if we get closer to goal
5: *n* ← *n* + 1 ⊳ increase planning horizon
6: $q_{k}^{*},\;d_{n,\text{min}}\leftarrow \underset{q}{\min}d_{n}\left(\bar{x},\Delta x\left(q\right)\right)$ ⊳ minimal distance to goal after *n* cycles
7: **end** **while**
8: $q_{1,k}^{*}\leftarrow -\text{sign}\left(q_{1,k-1}^{*}\right)q_{1,k}^{*}$ ⊳ switch sign of step length
9: ${\bar{\alpha }}_{k}\leftarrow \bar{\alpha }(q_{1,k}^{*},q_{2,k}^{*})$ ⊳ reference according to Eq. (14)
10: *f*_*k*_ ← ¬ *f*_*k* − 1_ ⊳ switch fixation
11: **return** ${\bar{\alpha }}_{k},\;f_{k}$ ⊳ next reference
12: **end procedure**

### 5.3. Experiments

**Figure 12:**
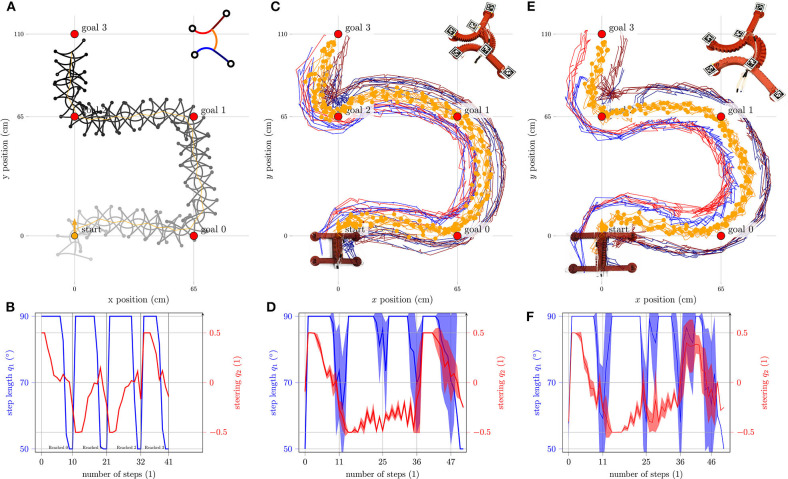
Simulation and experiment with the Gait Pattern Generator in action. In **(A)**, the simulation of gait for a list of four target positions is shown. In **(B)**, the course of *q*_1_ and *q*_2_ is plotted over the number of steps. In **(C,D)**, the corresponding plots are shown for the experiment with the small prototype. **(E,F)** Show the experiment with the large prototype. For the experiments, the color code is as follows: front left foot (red), front right foot (dark red), tip of the torso (orange), torso's end (dark orange), rear left foot (blue), and rear right foot (dark blue).

In Figure 12A, the simulation for a list of four different target positions is shown. The next target position becomes active when *d*_0_ < ϵ applies, i.e., the robot has almost reached the current target. In Figure 12B, the corresponding course of ***q*** is shown. It is clear to see that both values change over time. This proves that the robot can transition between different gait patterns, at least in the simulation. The same situation is now studied in the experiment shown in Figure 12C where the tracks of the tags of five independent experiments are overlaid. The difference between the right and left curves is significant. While the right-hand curves have a relatively small radius, the radii of the left-hand curves are much larger. This difference has already been noticed in the experiment from Figure 7; here, it is especially pronounced. The difference is due to manufacturing inaccuracies and pressure angle calibration, as discussed in section 4. The lacking ability to control the exact time of fixation of the feet also plays a role: since the strong actuation of a leg also deforms the suction cup, it may no longer be able to suck, despite negative pressure is applied. This effect is most prominent in the rear right foot. All other feet usually fix according to plan. However, the delayed fixation of the rear right leg supports a fast execution of the right turn (see Supplementary Video). Figure 12D shows the mean values and standard deviations of the step size *q*_1_ (blue) and the steering factor *q*_2_ (red). Here, the mean value was calculated over the number of steps. The different number of steps required results in a high standard deviation in the region of the four target positions. In order to reach the final target position, 45 steps were required in the fastest run and 51 steps in the worst run. The course of the mean value is similar to the simulation in Figure 12B and is not constant. Nevertheless, in all cases, the robot reaches the final goal and always follows a similar path. This proves that also the physical robot can transition between different gait patterns and the reproducibility of the experiments to a certain extent. Figures 12E,F show the results of the same experiment now performed with the robot from Seibel and Schiller (2018). The robot is basically the same, but is a little bigger (body length/span: 15/25). For the experiment, the same approximation of the direct kinematics was used (see Table 3), and still the robot shows the desired behavior. This shows that Δ*x* only needs to reflect the qualitative trend. The exact values are not particularly important because as Figures 12B,D,F show, the step length is most of the time at the maximum and therefore the goal cannot be reached within one cycle anyway.

## 6. Conclusion

The aim of this work was position control of the gecko-inspired soft robot from Schiller et al. (2019) in Cartesian space. The solution to this complex task is based on two major simplifications: (i) the formulation of a gait law to reduce the state space of the robot from nine to two dimensions and (ii) the approximation of the direct kinematics to allow a fast evaluation. The gait law restricts the choice of possible references extremely; e.g., only specific trotting gaits are allowed. In this work, it was successfully examined whether a position control system can function with this limitations. However, it has not been investigated whether a larger permitted choice of references leads to better results. In fact, it is possible that the introduction of additional generalized coordinates or a different gait law may lead to a better performance of the robot. Furthermore, neither frictional effects nor any dynamics were considered. Also, by approximating the direct kinematics in the polynomial Δ*x*, an assumption is made which is fulfilled only in very few cases (compare section 5.2). Instead of using the approximation, the simulation model could also be employed to find the best possible reference for the current situation. But the simulation of one step takes an average of 0.1 s on an *AM335x 1GHz ARM*Ⓡ *Cortex-A8* processor, which is used for control. With an average of 10 evaluations of the direct kinematics required to find the reference leading to the minimum distance, this adds up to 1 s. In contrast to a polynomial approximation where the Jacobi matrix can be easily formed to find the minimum efficiently, no analytical Jacobi matrix has been formulated for the simulation model so far. This means that when the simulation model is used, calculation would require most of the time of the cycle. However, the experiments show that the robot always reaches the target, even if the assumptions made in the derivation of the Gait Pattern Generator are not fulfilled and the approximation of the direct kinematics was done for a robot of different dimensions.

The path planning algorithm implemented is very basic, as it minimizes the Euclidean norm of the target vector, i.e., it dictates the direct path from the current position to the target. The gait law provides an intuitive way (forward and rotational speed) to control a quite complex robot and the approximation of the direct kinematics provides the resulting quantitative motion. This opens an interface to a wide variety of more dedicated path planning algorithms, as the robot can now be treated as a unicycle. For example, the path could be planned using Cartesian polynomials (Siciliano et al., 2010) and thus the robot orientation could also be controlled. Although the softness of the robot is very complex to model, it also allows the formulation of very drastic references, even if these cannot be fulfilled at all, as hindered by the closed kinematic chain. How these contradictory demands are solved is then “computed” by the body itself. Conventional parallel kinematic robots, such as the Stewart-Gough platform, would be damaged in this case. The gecko-inspired soft robot is therefore a good example of Embodied Intelligence (Cangelosi et al., 2015) or Morphological Computation (Pfeifer and Gómez, 2009) since it does the right thing “intuitively.” This is in agreement with the principle of controlling soft robots mainly in a feed forward way in order to maintain and make use of their softness (Santina et al., 2017). The cascaded controller structure, as discussed in the introduction, can therefore also be applied to position control of mobile robots. The method of deriving a basic locomotion strategy like the presented gait law by very simple (feet rotate only in swing phase), but mathematically (with the constant curvature model) unfulfillable assumptions, can be transferred to any other soft mobile robot. Although this needs to be done individually for each robotic platform, this work can serve as a reference for future and/or existing robots.

## Data Availability Statement

The raw data generated for this study are available on request.

## Author Contributions

LS derived the Gait Pattern Generator, performed the experiments, and discussed the results. LS and AS wrote and revised the manuscript. AS and JS supervised the project. All authors contributed to the article and approved the submitted version.

## Conflict of Interest

The authors declare that the research was conducted in the absence of any commercial or financial relationships that could be construed as a potential conflict of interest.
