# Supplementary material for: A Gait Pattern Generator for Closed-Loop Position Control of a Soft Walking Robot
Source: Front Robot AI. 2020 Jul 2;7:87. doi: 10.3389/frobt.2020.00087 (PMC7806028; doi:10.3389/frobt.2020.00087)
Supplement: Supplementary file 1 [file Data_Sheet_1.pdf]

A gait pattern generator for closed-loop position control of a soft walking robot – Supplementary Material

Lars Schiller *, Arthur Seibel and Josef Schlattmann

Saturday 20th June, 2020

Contents

1 Brief description of experimental setup **2**

2 Suction cup experiments **4**

3 Determination of additional bending for fixed legs **4**

4 Methods for data processing **8**

5 Identification of the factors that influence motion **13**

6 Processing steps for first experiment **14**

7 Processing steps for second experiment **14**

8 Influence of model order of fitting polynomial **15**

9 Calibration Procedure **15**

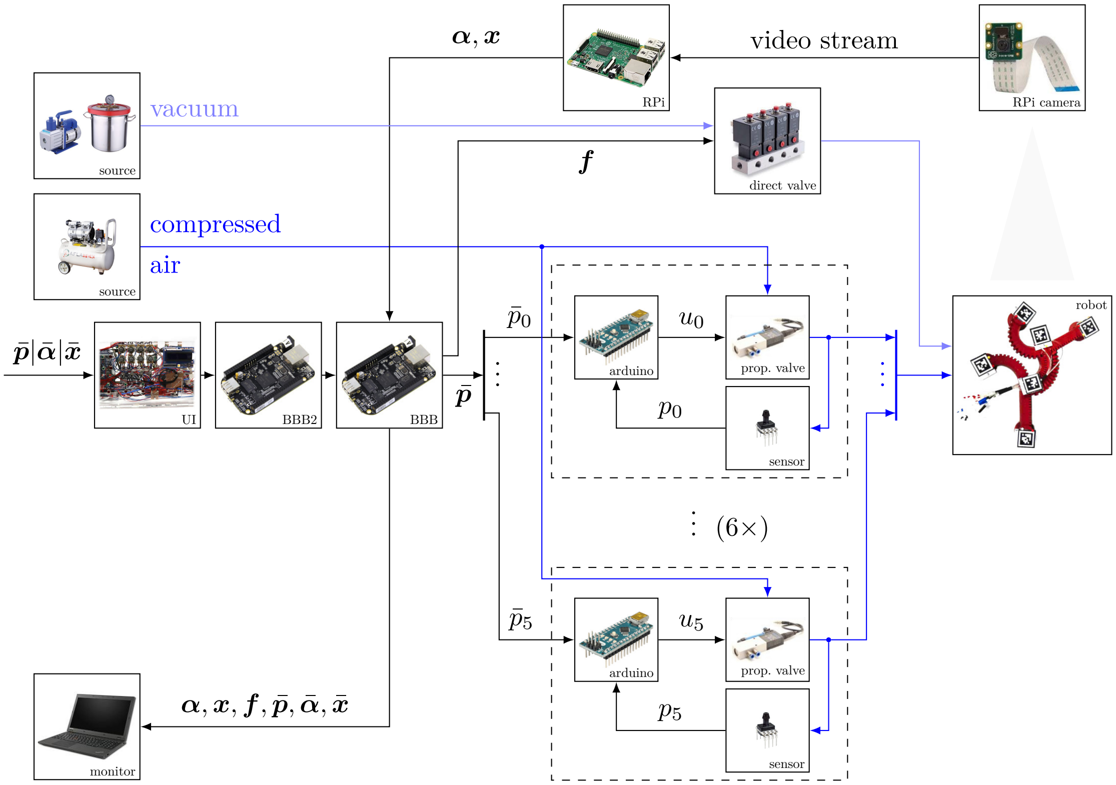

Figure 1: Diagram of main hardware components used for controlling and evaluating the robot. Therein $\bar{p}_i$ denotes the pressure reference for the i-th channel, $\bar{\alpha}_i$ the angular reference for the i-th channel, u_i the control signal of the i-th proportional valve, $\boldsymbol{\alpha}$ the vector containing the angle measurements, $\boldsymbol{x}$ the vector containing the position measurements, and $\boldsymbol{f}$ the vector containing control signals for the direct-acting solenoid valves, i.e. the fixation states of the feet. UI is short for "User Interface Unit", BBB is an abbreviation for BeagleBone Black, i.e., the single-board computer used in the control box and RPi is short for Raspberry Pi, i.e., the single-board computer used in the measurement system.

1 Brief description of experimental setup

Figure 1 shows a diagram of the main hardware components installed in the experimental setup which was used in the paper. With the camera system and the python-package `apriltag`, the center and corners of the tags can be measured in pixel coordinates. The positions of the corner points can be used to determine the orientation of the individual tags relative to the camera coordinate system:

$$\{\boldsymbol{x}_i\}_O = \frac{\boldsymbol{p}_{i,1} - \boldsymbol{p}_{i,0}}{|\boldsymbol{p}_{i,1} - \boldsymbol{p}_{i,0}|}\,, \qquad (1) \qquad\qquad \{\boldsymbol{y}_i\}_O = \frac{\boldsymbol{p}_{i,0} - \boldsymbol{p}_{i,3}}{|\boldsymbol{p}_{i,0} - \boldsymbol{p}_{i,3}|}\,. \qquad (2)$$

where $\boldsymbol{p}_{i,j}$ is the vector pointing to the jth tag corner of the ith tag described in the camera fixed coordinate system.

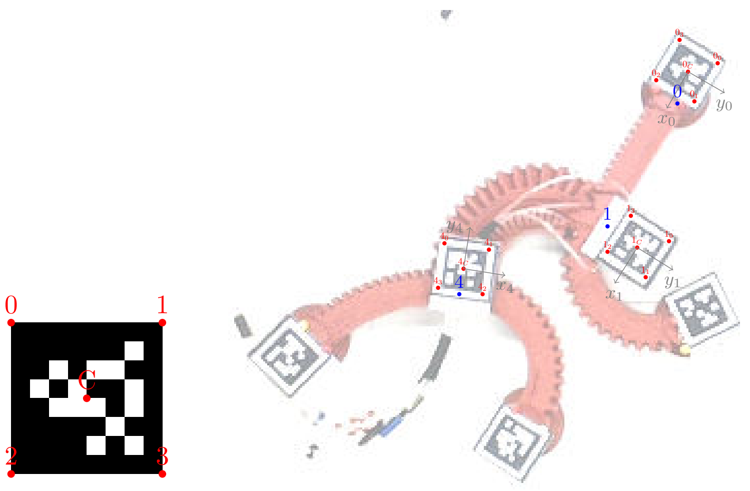

(a) (b)

Figure 2: Visualization of the shifted position measurement. (a) Shows the center point and corner points of a single april tag. (b) Shows how the shift happens to tag 0, 1, 4.

Table 1: Shift of position measurements and mapping between tags and actuators.

Shift of position measurements						
Tag ID	0	1	2	3	4	5
x-shift s_x [px]	10	0	−10	10	0	−12
y-shift s_y [px]	5	−20	5	−5	−9	−5

Mapping between tags and actuators						
Actuator ID	0	1	2	3	4	5
left tag	0	1	1	4	4	5
right tag	1	2	4	1	3	4

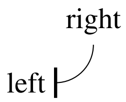

1.1 Position measurement

Since the tags cannot be placed exactly where the measurement is to be made, the raw measurement is shifted. Table 1 summarizes the shift. The position of the shifted tags is then described as

$$\{\boldsymbol{p}_i\}_O = \boldsymbol{p}_{i,C} + s_{x_i}\{\boldsymbol{x}_i\}_O + s_{y_i}\{\boldsymbol{y}_i\}_O \,. \tag{3}$$

Figure 2 illustrates the shift of the measurement in order to obtain the position corresponding to the simulation model.

1.2 Angle measurement

In order to determine the angle of an actuator, the orientation of the two tags at one end and at the other is compared. In order to achieve the highest possible resolution, the diagonal is chosen to determine the orientation vector $\boldsymbol{o}$ (corner points 0 and 2):

$$\{\boldsymbol{o}_i\}_O = \boldsymbol{p}_{i,2} - \boldsymbol{p}_{i,1} \,. \tag{4}$$

The angle of an actuator is then determined by comparing the left and right tag. The left side indicates the side that is clamped when the actuator is bent by 90 degrees and arranged as shown in Table 1. The table also shows which tags are

left and right for which actuator. The angle can then be calculated as follows:

$$\alpha_i = \tilde{\alpha}(\boldsymbol{o}_{i,\text{left}}, \boldsymbol{o}_{i,\text{right}}) \tag{5}$$

$$\tilde{\alpha}(\boldsymbol{o}_1, \boldsymbol{o}_2) = \begin{cases} \tilde{\boldsymbol{o}}_2 = \boldsymbol{R}(-\operatorname{atan2}(o_{y,1}, o_{x,1}) - \varphi_\text{B})\boldsymbol{o}_2 \\ \tilde{\alpha} = \operatorname{atan2}(\tilde{o}_{y,2}, \tilde{o}_{x,2}) + \varphi_\text{B} \end{cases} \tag{6}$$

with $\boldsymbol{R}$ as rotation matrix around the z axis. The orientation angle of the robot is calculated as follows:

$$\varepsilon = \tilde{\alpha}(\boldsymbol{e}_x, \boldsymbol{p}_1 - \boldsymbol{p}_4) \ . \tag{7}$$

2 Suction cup experiments

Figure 3 shows the test set-up for determining the normal pull-off force, as well as the pull-off force in the transverse direction of the suction cup. The experimental results are also shown. For determining the normal pull-off force, a constant vacuum was applied and the additional weight was slowly increased until the pull-off event happens. To determine the shear pull-off force, the additional weight was set constant and the applied vacuum was varied. For this purpose a venting valve was added to the setup. For each measurement point, the acrylic glass was cleaned with isopropanol before attaching the suction cup.

3 Determination of additional bending for fixed legs

3.1 Via simulation

```
GeckoBotModel/Scripts/finding_gait_law/analytic_model_5.py
GeckoBotModel/Scripts/finding_gait_law/analytic_model_5_ref_simmodel.py
GeckoBotModel/Scripts/finding_gait_law/analytic_model_5_ref_simmodel_(89,10,5_9).py
```

For a constant step length q_1, the inner stress was simulated for varying steering q_2 and varying additional bending c_1. The optimal c_1 seems to proportionally depend on the step length q_1 if stress is to be minimized. The first row in Figure 4 shows the simulation results for the parameter set used in (Schiller et al., 2020). With c_1, the rotation of the robot per cycle $\Delta\varepsilon$ also grows. So, if the rotation is to be maximized, a rather high c_1 is advantageous. However, a higher c_1 also creates more stress. For high steering factors q_2, the simulation will quickly go into the red area. Since the robot should run at full speed most of the time, $c_1 = 1$ is chosen for this parameter set, for moderate stress during movement with large step lengths q_1, and high rotation $\Delta\varepsilon$ per cycle for all step sizes. The determination of c_1 was repeated with a modified simulation model ($f_l, f_o, f_a = 0.2, 12.1, 6.1$). The second row in Figure 4 shows the result. Note that heat map runs from 0 to 1500 this time (and not from 0 to 250 as before). With this parameter set, $c_1 = 0.4$ is chosen, because this minimizes the stress for large step lengths. The third row in Figure 4 shows the result for simulation parameters $f_l, f_o, f_a = 89, 10, 5.9$, the parameter set used in the paper. With this parameter set, also $c_1 = 0.4$ is chosen. Table 2 summarizes the optimal c_1 (with respect to inner stress) for different simulation parameters and step lengths.

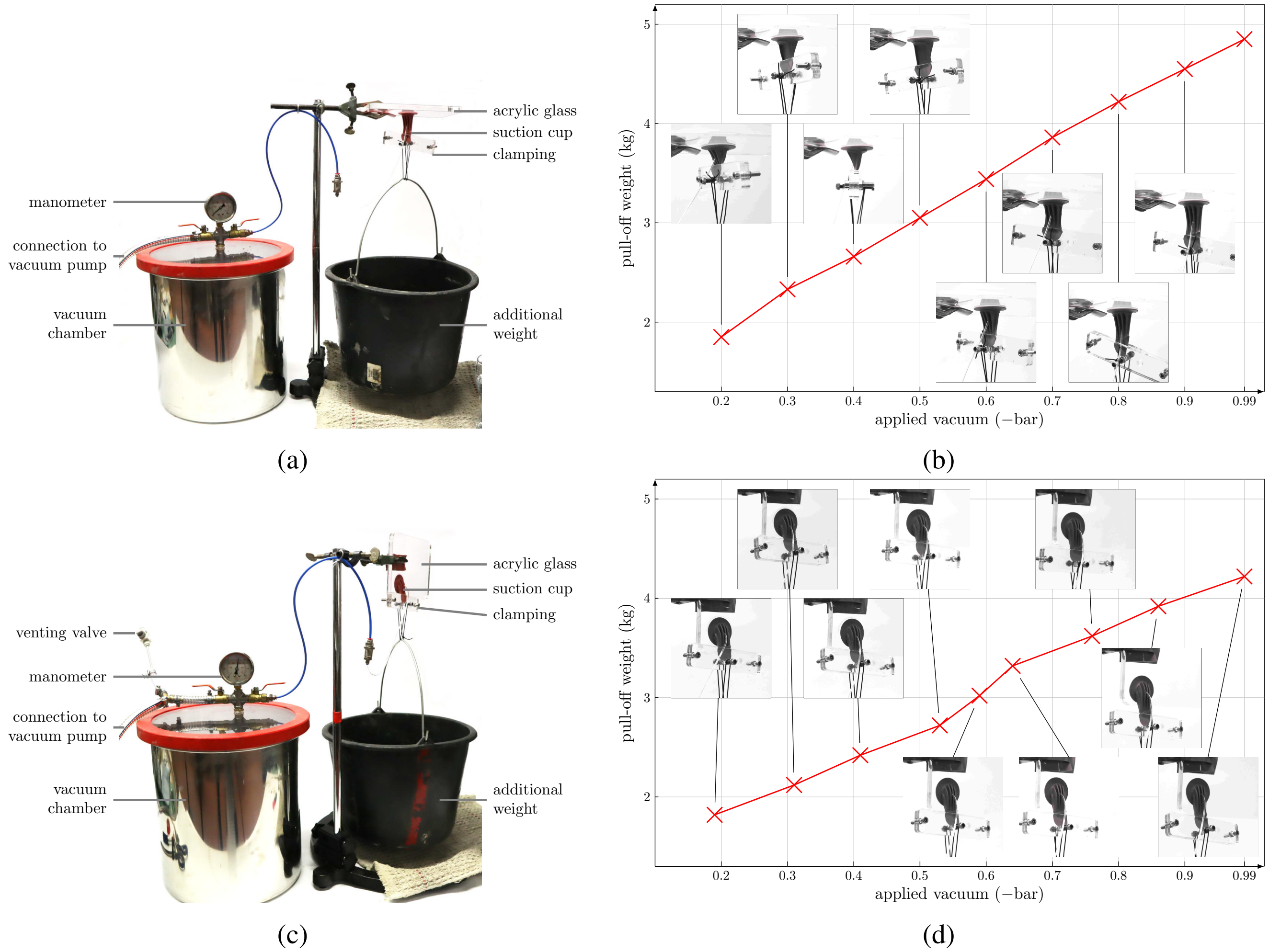

Figure 3: Experiments for determining the pull-off force of the suction cup. (a) Experimental setup for determining the pull-off normal force and (b) experimental results. (c) Experimental setup for determining the pull-off shear force and (d) experimental results (for each measurement point, the acrylic glass was cleaned with isopropanol before attaching the suction cup).

Table 2: Resulting additional bending angles for fixed legs c_1 for different simulation parameters.

f_l, f_o, f_a	60	70	80	90
0.1, 1, 10	< 0.6	~ 0.6	~ 0.7	~ 0.9
0.2, 12, 6	~ 0.2	~ 0.2	~ 0.3	~ 0.4
89, 10, 5.9	~ 0.2	~ 0.2	~ 0.3	~ 0.4

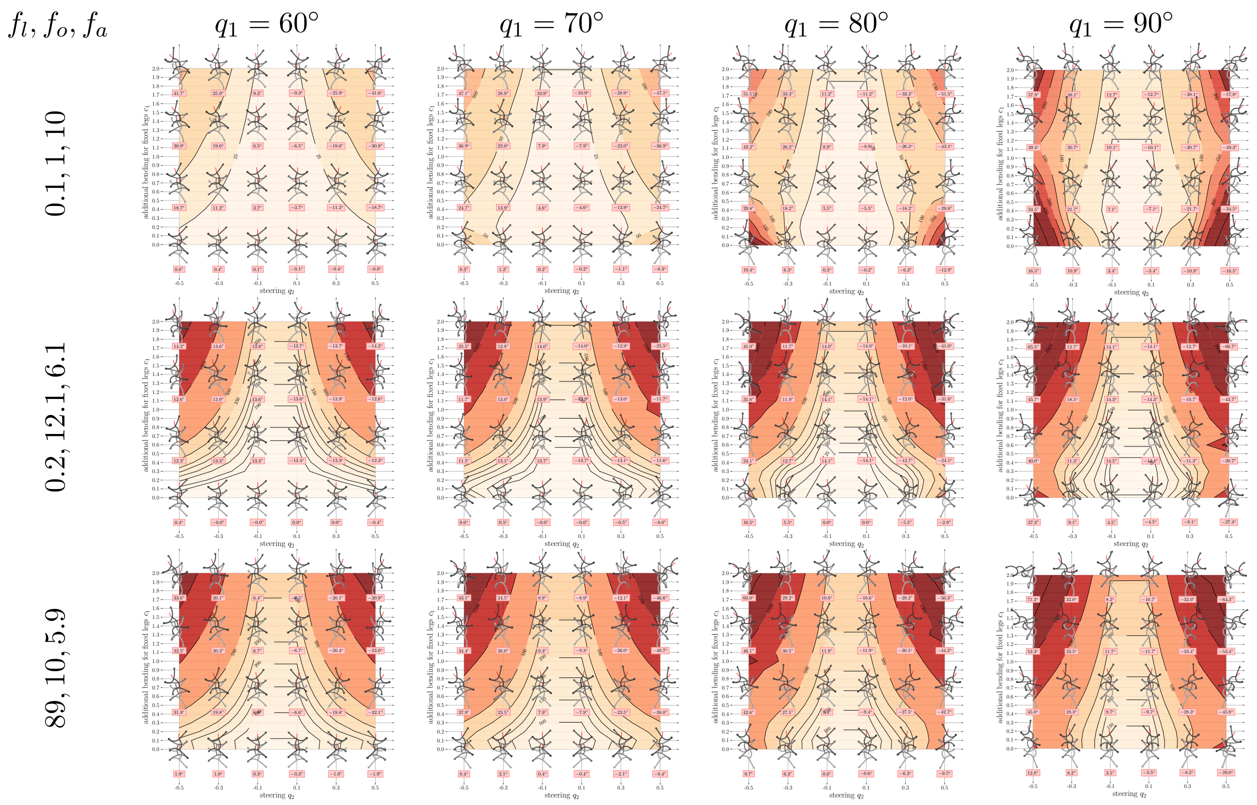

Figure 4: Simulation of the influence of additional bending for fixed feet c_1 on stress and body rotation for different step lengths q_1 and different simulation weightings f_l, f_o, f_a.

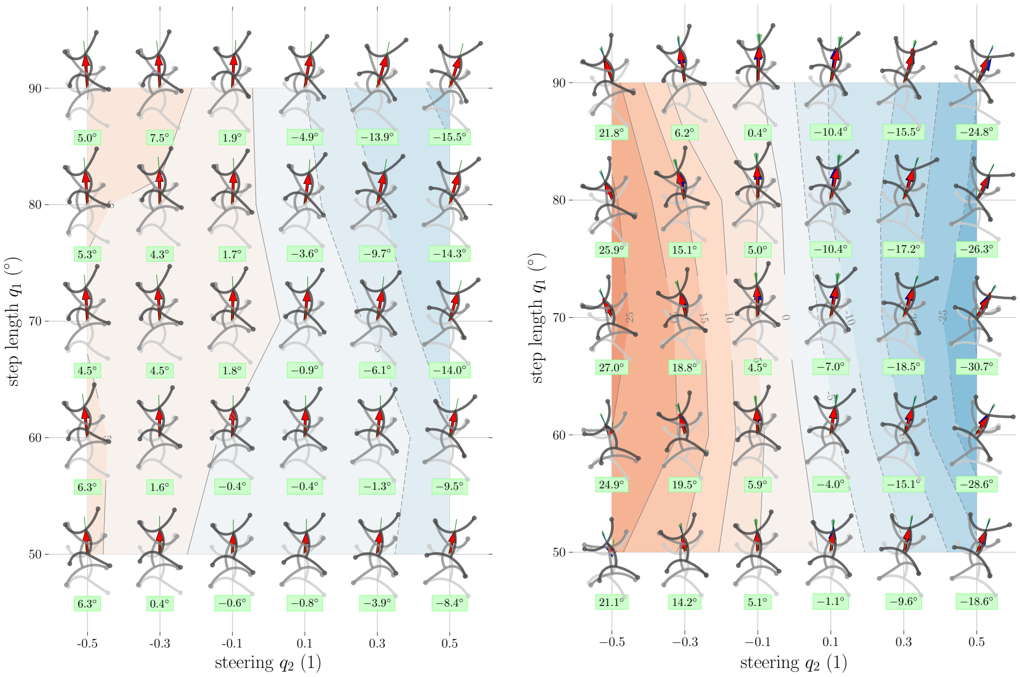

(a) $c_1 = 0.4$ (b) $c_1 = 1.0$

Figure 5: Experimental analysis of the influence of additional bending for fixed legs c_1 on body rotation for different step lengths q_1. (a) $c_1 = 0.4$, (b) $c_1 = 1.0$.

3.2 Via experiment

`GeckoBotExperiments/2020_01_GaitLawExp/eval_c104.py`
`GeckoBotExperiments/2020_01_GaitLawExp/eval_c110_redo.py`

The resulting motion for different values of c_1 was determined experimentally for the entire configuration space. As it turns out, $c_1 = 0.4$ leads to much less rotation than $c_1 = 1.0$ (compare Fig. 5). Therefore, $c_1 = 1$ is used in the paper.

4 Methods for data processing

`GeckoBotExperiments/2019_12_GaitLawExp/main_eval.py`
`GeckoBotModel/Scripts/finding_gait_law/analytic_model_6_compare_to_exp.py`

Here, methods are defined which are used for the evaluation of the experiments carried out in the paper.

4.1 Correction of measurement

The pre-processed raw data can now be checked for plausibility. Here, we try to slightly change the measured angles and positions so that they correspond as closely as possible to the model conception of specially arranged circular arcs. This optimization is based on the basic idea that the lengths of the individual limbs are not variable in length. First, the double angle measurement of the torso is removed. This simplifies the handling of the double channel, but it is a hindrance for the calculation in angular coordinates

$$\boldsymbol{\alpha} = [\alpha_0 \; \alpha_1 \; \alpha_2 \; \alpha_4 \; \alpha_5] \; . \tag{8}$$

Next, the lengths of the limbs must be defined and combined into a vector:

$$\boldsymbol{\ell} = [\ell_l \; \ell_l \; \ell_t \; \ell_l \; \ell_l] \; , \tag{9}$$

where $\ell_l = 79\,\text{px}$ is the length of a leg and $\ell_t = 92\,\text{px}$ is the length of the torso (camera resolution: $1648 \times 1232\,\text{px}^2$). Now, deviations of the correction and the measurement are allowed within certain limits. The angles as well as the measured positions of the feet/torso may differ. For this reason, the vector $\boldsymbol{\beta} = [\beta_0 \; \beta_1 \; \beta_2 \; \beta_3 \; \beta_4]$ is defined, which describes the angular deviation. The corrected bending angles are then defined as

$$\hat{\boldsymbol{\alpha}} = \boldsymbol{\alpha} + \boldsymbol{\beta} \; . \tag{10}$$

The position of the front torso end is also set as variable:

$$\hat{\boldsymbol{p}}_1 = [p_{1,x} \; p_{1,y}] \; . \tag{11}$$

The quantity to be optimized is then defined as $\boldsymbol{m}$ (for measurement):

$$\boldsymbol{m} = [\hat{\boldsymbol{p}}_1 \; \boldsymbol{\beta} \; \hat{\varepsilon}] \in \mathbb{R}^8 \; , \tag{12}$$

where $\hat{\varepsilon}$ describes the orientation of the robot to be optimized. The corrected positions $\hat{\boldsymbol{P}}$ then result in

$$\hat{\boldsymbol{P}} = \tilde{\boldsymbol{P}}(\boldsymbol{\ell}, \hat{\boldsymbol{\alpha}}, \hat{\varepsilon}, \hat{\boldsymbol{p}}_1) \; , \tag{13}$$

where the function $\tilde{\boldsymbol{P}}$ for calculating the positions is introduced in Section 4.2.

The quality of the correction can then be quantified using the following function:

$$\sigma(\boldsymbol{m}) = w_x ||\hat{\boldsymbol{P}} - \boldsymbol{P}||_2 + w_\alpha |\boldsymbol{\beta}|_2 + w_\varepsilon |\hat{\varepsilon} - \varepsilon|_2 \; . \tag{14}$$

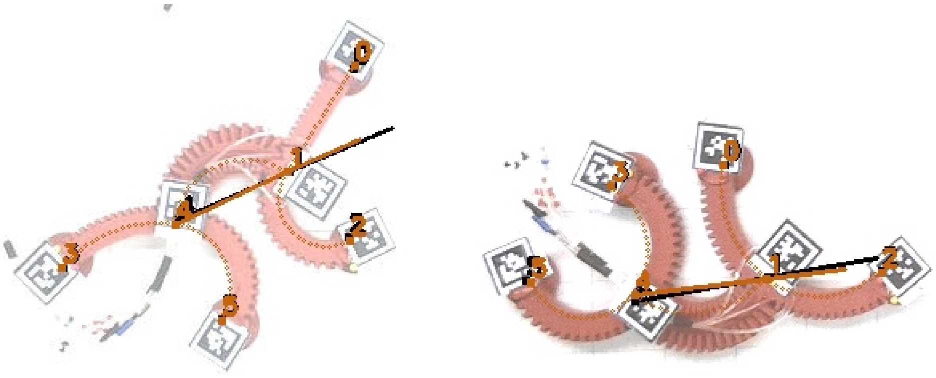

(a) small correction (b) bigger correction

Figure 6: Visualization of measurements correction.

Here, w_x denotes the weighting factor for the deviation of the corrected positions to the measured ones, w_α the weighting factor for the deviation of the bending angles, and w_ε the weighting for the deviation of the robot orientation. The optimal correction then results as the minimum of the quality functional:

$$\boldsymbol{m}^* = \min_{\boldsymbol{m}} \sigma(\boldsymbol{m}) \ . \tag{15}$$

Figure 6 illustrates the correction algorithm. The (pre-processed shifted) raw data are shown in black and the corrections are shown in orange. Depending on the pose, much or little correction is made.

4.2 Calculation of feet positions

General transformation matrices:

$$\tilde{\boldsymbol{T}}(\varepsilon, \boldsymbol{p}) = \begin{bmatrix} \cos(\varepsilon) & -\sin(\varepsilon) & p_x \\ \sin(\varepsilon) & \cos(\varepsilon) & p_y \\ 0 & 0 & 1 \end{bmatrix} , \tag{16}$$

$$\boldsymbol{T}(\alpha, \ell) = \begin{bmatrix} \cos(\alpha) & -\sin(\alpha) & \frac{\ell}{\alpha}\sin(\alpha) \\ \sin(\alpha) & \cos(\alpha) & \frac{\ell}{\alpha}(1-\cos(\alpha)) \\ 0 & 0 & 1 \end{bmatrix} . \tag{17}$$

Transformation matrices:

$$^{O}\boldsymbol{T}_1 = \tilde{\boldsymbol{T}}\left(\varepsilon - \frac{\alpha_2}{2}, \boldsymbol{p}_1\right) , \tag{18}$$

$$^{1}\boldsymbol{T}_0 = \boldsymbol{T}(-\alpha_0, \ell_0) , \tag{19}$$

$$^{1}\boldsymbol{T}_2 = \boldsymbol{T}(\alpha_1, \ell_1) , \tag{20}$$

$$^{1}\boldsymbol{T}_4 = \boldsymbol{T}(\pi, 0)\, \boldsymbol{T}(\alpha_2, \ell_2) , \tag{21}$$

$$^{4}\boldsymbol{T}_3 = \boldsymbol{T}\left(-\frac{\pi}{2}, 0\right) \boldsymbol{T}(\alpha_3, \ell_3) , \tag{22}$$

$$^{4}\boldsymbol{T}_5 = \boldsymbol{T}\left(\frac{\pi}{2}, 0\right) \boldsymbol{T}(-\alpha_4, \ell_4) . \tag{23}$$

Positions of feet:

$$^{O}\boldsymbol{p}_0 = {}^{O}\boldsymbol{T}_1\, {}^{1}\boldsymbol{T}_0\, \boldsymbol{e}_z \ . \tag{24}$$

$$^{O}\boldsymbol{p}_2 = {}^{O}\boldsymbol{T}_1\, {}^{1}\boldsymbol{T}_2\, \boldsymbol{e}_z \ , \tag{25}$$

$$^{O}\boldsymbol{p}_3 = {}^{O}\boldsymbol{T}_1\, {}^{1}\boldsymbol{T}_4\, {}^{4}\boldsymbol{T}_3\, \boldsymbol{e}_z \ , \tag{26}$$

$$^{O}\boldsymbol{p}_4 = {}^{O}\boldsymbol{T}_1 \, {}^{1}\boldsymbol{T}_4 \, \boldsymbol{e}_z \; , \qquad (27)$$

$$^{O}\boldsymbol{p}_5 = {}^{O}\boldsymbol{T}_1 \, {}^{1}\boldsymbol{T}_4 \, {}^{4}\boldsymbol{T}_5 \, \boldsymbol{e}_z \; . \qquad (28)$$

where $\boldsymbol{e}_z$ is the unit vector in z-direction. The matrix $\tilde{\boldsymbol{P}}$ now combines all position vectors:

$$\tilde{\boldsymbol{P}} = [\boldsymbol{p}_0 \; \boldsymbol{p}_1 \; \boldsymbol{p}_2 \; \boldsymbol{p}_3 \; \boldsymbol{p}_4 \; \boldsymbol{p}_5]^\top = [\boldsymbol{p}_x \; \boldsymbol{p}_y] \in \mathbb{R}^{6\times 2} \; . \qquad (29)$$

4.3 Time shift operator

In order to be able to compare the data from the individual experiments with each other, the time stamp is shifted. First, the time stamp is determined, where the front left foot is fixed for the very first time:

$$t_0 = t_k \quad \text{if} \quad f_0(t_k) = 1 \; . \qquad (30)$$

Then, every time stamp is subtracted by t_0:

$$t_k = t_k - t_0 \; . \qquad (31)$$

4.4 Rotation operator

The orientation of the robot is rotated such that all measurement series initially run in the same direction. First, the robot orientation at the initial time stamp is determined:

$$\varepsilon_0 = \varepsilon(t_0) \; . \qquad (32)$$

Then the whole measurement is shifted:

$$\varepsilon_k = \varepsilon_k - \varepsilon_0 \; . \qquad (33)$$

Accordingly, the measured positions must be rotated:

$$\begin{bmatrix} x_k \\ y_k \end{bmatrix} = \boldsymbol{R}(-\varepsilon_0) \begin{bmatrix} x_k \\ y_k \end{bmatrix} \; . \qquad (34)$$

In addition, the position are shifted, such that all measurement series start from the same Cartesian point:

$$\begin{bmatrix} x_{1,0} \\ y_{1,0} \end{bmatrix} = \begin{bmatrix} x_{1,k} \\ y_{1,k} \end{bmatrix} (t_0) \; , \qquad (35)$$

$$\begin{bmatrix} x_k \\ y_k \end{bmatrix} = \begin{bmatrix} x_k \\ y_k \end{bmatrix} - \begin{bmatrix} x_{1,0} \\ y_{1,0} \end{bmatrix} , \qquad (36)$$

where $(x_{1,0}, y_{1,0})$ is the vector pointing to the position of the torso's front at time t_0.

4.5 Finding extreme poses

The samples corresponding to an extreme pose are searched for. An extreme pose is recognized by the fact that the pressure reference r_k changes in the next sample. These samples are packed into a vector which contains only the samples of the extreme poses and is therefore indexed with EP:

$$\varepsilon_{\mathrm{EP}} = \varepsilon_k \ \text{ if}: \ r_{k+1} \neq r_k \ . \tag{37}$$

4.6 Determination of robot's orientation

The orientation of the robot is calculated by the tag positions:

$$\hat{\alpha}(\boldsymbol{r}_1, \boldsymbol{r}_2) = \begin{cases} \varphi_1 = \operatorname{atan2}(r_{y,1}, r_{x,1}) \\ \tilde{\boldsymbol{r}}_2 = \boldsymbol{R}(-\varphi_1)\boldsymbol{r}_2 \\ \hat{\alpha} = \operatorname{atan2}(\tilde{r}_{y,2}, \tilde{r}_{x,2}) \end{cases} , \tag{38}$$

$$\varepsilon = \hat{\alpha}(\boldsymbol{e}_x, \boldsymbol{p}_1 - \boldsymbol{p}_4) \ . \tag{39}$$

4.7 Determination of change of robot's orientation per cycle

The change in orientation per cycle can be determined by the derivative of the vector containing the orientation of the extreme positions with respect to the samples. The derivative is multiplied by two, since a cycle consists of two extreme poses:

$$\Delta\varepsilon = 2\frac{\mathrm{d}}{\mathrm{d}n}\varepsilon_{\mathrm{EP}} \ . \tag{40}$$

Another possibility is to derivate the vector with respect to time. Both operations lead to the same results, because the poses are taken in regular time intervals. The derivative with respect to time can be determined by a first order least squares polynomial fit. This also provides the shift c:

$$\dot{\varepsilon}^*, c^* = \min \sum_{k=0}^{N} |\dot{\varepsilon} t_{\mathrm{EP},k} + c - \varepsilon_{EP,k}| \ , \tag{41}$$

where N describes the number of extreme poses within one measurement. With the two optimized parameters $\dot{\varepsilon}^*$ and c^*, a first-order polynomial can be created:

$$\hat{\varepsilon}(t) = \dot{\varepsilon}^* t + c^* \ . \tag{42}$$

The relationship between the two calculation variants for the change of orientation of the robot is as follows:

$$\Delta\varepsilon = \frac{2\dot{\varepsilon}^*\Delta t}{N-1} \ , \tag{43}$$

where $\Delta t = t_N - t_0$ describes the complete duration of the measurement and t_N describes the time where pose N is taken by the robot. Figure 7 shows the course of the orientation of the robot during 15 different experiments. In addition, the polynomial $\hat{\varepsilon}$ resulting from the least-square-fit is shown.

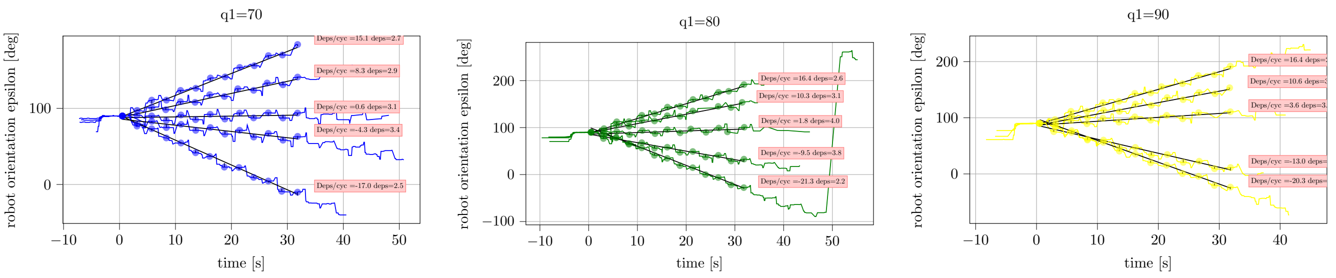

(a) $q_1 = 70°$ (b) $q_1 = 80°$ (c) $q_1 = 90°$

Figure 7: Determination of change of robot's orientation per cycle $\Delta\varepsilon$ and the amplitude of robot's oscillation around trend line.

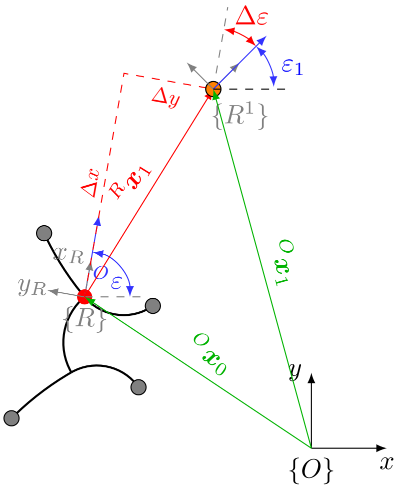

Figure 8: Visualization of Eq. (44).

4.8 Determination of distance traveled

Since the robot-fixed coordinate system changes with each new pose with respect to the spatial-fixed coordinate system, determining the displacement vector is not trivial. This means that the displacement from pose 0 to pose 2 cannot be compared to the displacement from pose 2 to pose 4 without transformation. Since the measurements are not complete in every pose due to smeared images, i. e., they contain empty samples, we want to use as many measurements as possible to determine the displacement vector $\Delta\boldsymbol{x}$. In order to compare the displacements between the cycles, they can be transformed into the fixed coordinate system with the following formula:

$$\Delta\boldsymbol{x} = {}^{R_k}\boldsymbol{x}_k = \boldsymbol{R}(-{}^{O}\varepsilon_k)\left({}^{O}\boldsymbol{x}_{k+1} - {}^{O}\boldsymbol{x}_k\right) , \tag{44}$$

where k describes the index of the kth cycle (not pose; 1 cycle = 2 poses). This allows to compare the resulting motion between all measured cycles. Figure 8 illustrates this formula.

5 Identification of the factors that influence motion

5.1 Initial pose

The resulting motion of the robot depends directly on how it is initially placed on the walking plane. Basically, two initial poses are useful:

1. For all experiments/simulations, always choose the same initial pose, e. g., the pose according to the law for straight gait with a step length of $q_1 = \frac{\pi}{2}$ $\left(\rightarrow \boldsymbol{\alpha}_0 = \bar{\boldsymbol{\alpha}}_{\text{straight}}(\frac{\pi}{2})\right)$. In this way, in addition to the movement, the ability of transitioning to another gait would also be examined at the same time. Or
2. choose always exactly the initial pose that corresponds to the current working point $(\rightarrow \boldsymbol{\alpha}_0 = \bar{\boldsymbol{\alpha}}_0(q_1, q_2))$. Thus, only the influence of the reference is examined and not other phenomena.

Since at this point the focus is on the resulting movement for the individual references and not on the feasibility of switching between different gaits, the second option is chosen for the initial pose.

5.2 Number of cycles

It is assumed that the same shift in position or rotation is generated in each cycle. Therefore, it is theoretically sufficient to measure one cycle. However, the behaviour may change in the second cycle. This happens if the reference angles cannot be fulfilled and consequently internal stress is generated. From the second cycle onwards, a certain balance is established and the gait becomes stable (in the sense that subsequent poses are mirror images of the current pose). It is therefore advisable to measure at least two cycles and drop the initial cycle.

5.3 Dimensions of robot

The lengths of the limbs have a significant influence on the shift in position. In order to be able to compare simulation and experiment better, the lengths of the limbs in the simulation are chosen as the actual lengths. The numerical values from the simulation can then be compared directly with the values from the experiment.

5.4 Weighting parameters (simulation-specific)

The weighting parameters have a huge influence on the simulation. They determine how the robot behaves for the given input. They are chosen to best match the simulation and the experiment.

5.5 Model order of fitting polynomial

The resulting motion of the robot for each pair of (q_1, q_2) shall be approximated with a polynomial of the form

$$\Delta \boldsymbol{x}(q_1, q_2) = \begin{bmatrix} \Delta\varepsilon \\ \Delta x \\ \Delta y \end{bmatrix} \ , \ \ \Delta\varepsilon, \Delta x, \Delta y := \sum_{i,j} a_{i,j} q_1^i q_2^j \ . \tag{45}$$

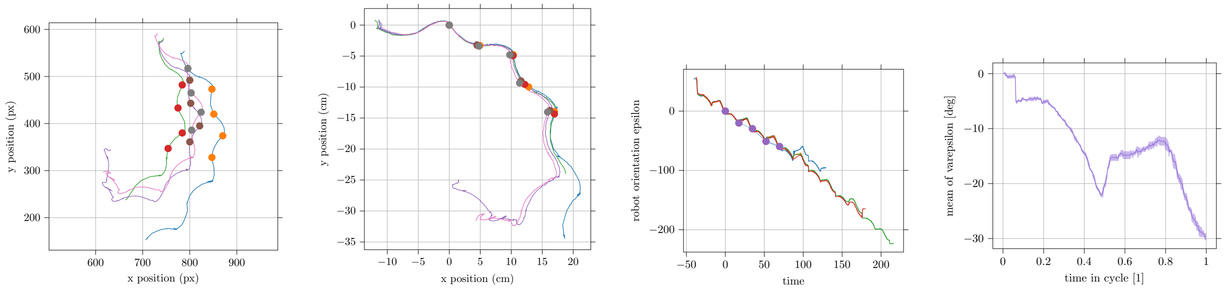

(a) raw data (b) rotated/shifted (c) extracted quantity (d) mean value

Figure 9: Visualization of processing steps for the first experiment.

The higher the model order, the better the fit matches the gridded measurement points. But this also increases the complexity and thus the evaluation time. Here, the question arises how exactly the fit has to match at all. The Supplementary Material contains extracts of a study to evaluate the influence of order. Since a simple model is desired, order 2 is chosen.

6 Processing steps for first experiment

`GeckoBotExperiments/2020_01_GaitLawExp/eval_c110_redo_slow.py`

The following processing steps were performed in paper on the measurement data from the first experiment:

1. Load raw data from independently performed experiments into the memory (Fig. 9 (a)).
2. Finding the extreme positions (in Fig. 9 (a) represented as dots).
3. Shift and rotate the raw data such that the position and orientation of the robot is always the same at the end of the first cycle (Fig. 9 (b)).
4. The first cycle is dropped since the initial cycle is always a bit different than all following cycles.
5. Extract the quantity to be examined from the transformed measurement data (in Fig. 9 (c), this is exemplary shown for the robot orientation ε).
6. Determine the mean value and standard deviation over all experiments for each point in time (in Fig. 9 (d), this is exemplary shown for the robot orientation ε).

7 Processing steps for second experiment

`GeckoBotExperiments/2020_01_GaitLawExp/eval_c110_redo.py`

As this experiment was performed at full speed, the measurement data show much more missing data due to undetected tags. In order to deal with this and also to increase the accuracy, different measurement series are again superimposed and averaged. In contrast to the first experiment, the averaging is not done over independently performed experiments, but over all cycles of one experiment. This simplifies the execution of the experiments considerably. The procedure is basically

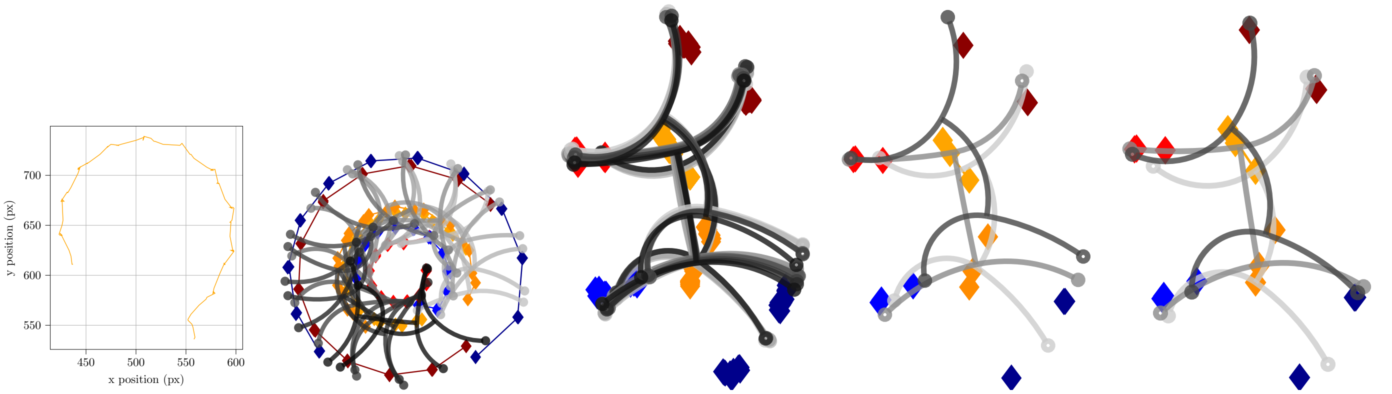

(a) raw data (b) extract (c) transform (d) average (e) correct

Figure 10: Visualization of processing steps for the second experiment.

carried out according to Eq. (44) and is visualized in Figure 10. The following processing steps were performed in the paper on the measurement data from the second experiment:

1. Load the raw data for an experiment and filter how many extreme poses the gait has (`POSE_IDX`) (in Fig. 10 (a) shown as dots).

2. Extract the next three poses for each beginning of the cycle `idx in range(POSE_idx[:-3], 2)` (in Fig. 10 (b), all measured extreme poses are shown)

3. Move and rotate these three poses so that they start from origin $(0, 0)$ and point in the initial direction (compare Fig. 10 (c)).

4. Save all relevant information to temporary memory (foot positions `fpos`, state vector `x`, fixation state `fix`).

5. Calculate the average value from the temporary memory (Fig. 10 (d)).

6. Correct the poses from the averaged data (`inverse_kinematics.py`) as described in Section 4.1 (Fig. 10 (e)).

8 Influence of model order of fitting polynomial

`GeckoBotModel/Scripts/finding_gait_law/analytic_model_6.py`.

Figure 11 shows a heat map of the fitting error for different model orders of the bivariate polynomial $\Delta\boldsymbol{x}(\boldsymbol{q})$. Since there is not much difference between order 2 (mean error of 9.5 mm) and order 4 (mean error of 4 mm), a model order of 2 is chosen in the paper.

9 Calibration Procedure

Each method of calibration results in a different alpha-pressure curve. Therefore, we try to adapt the calibration procedure to the real operating conditions as far as possible. The procedures listed in Table 3 were carried out. Figure 12 shows the

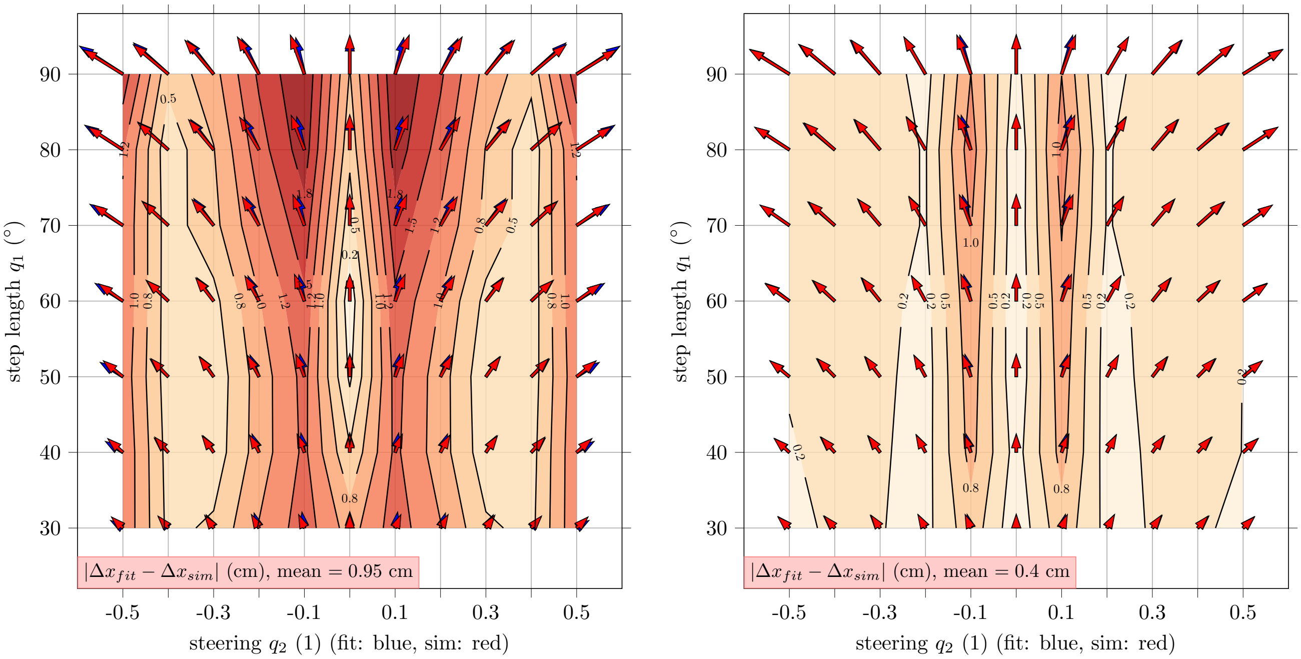

(a) order = 2

(b) order = 4

Figure 11: Analysis of model order of the bivariate polynomial $\Delta \boldsymbol{x}(\boldsymbol{q})$. Mean error of order 2 is 9.5 mm and mean error of order 4 is 4 mm.

qualitative course of pressure references for the different procedures and Figure 13 shows the resulting α-p curves.

References

Schiller, L., Seibel, A., and Schlattmann, J. (2020). A lightweight simulation model for soft robot's locomotion and its application to trajectory optimization. *IEEE Robotics and Automation Letters* 5, 1199–1206

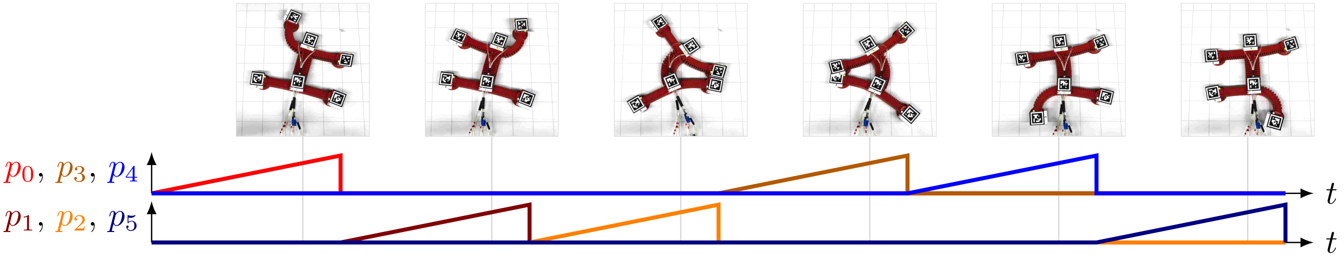

(a) mode 1

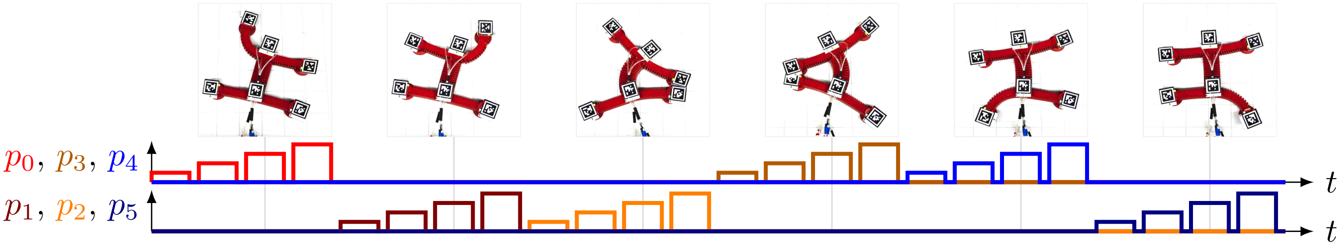

(b) mode 2

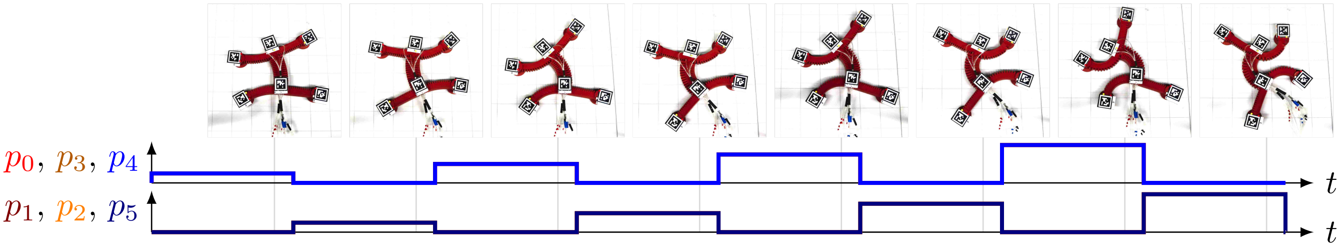

(c) mode 3

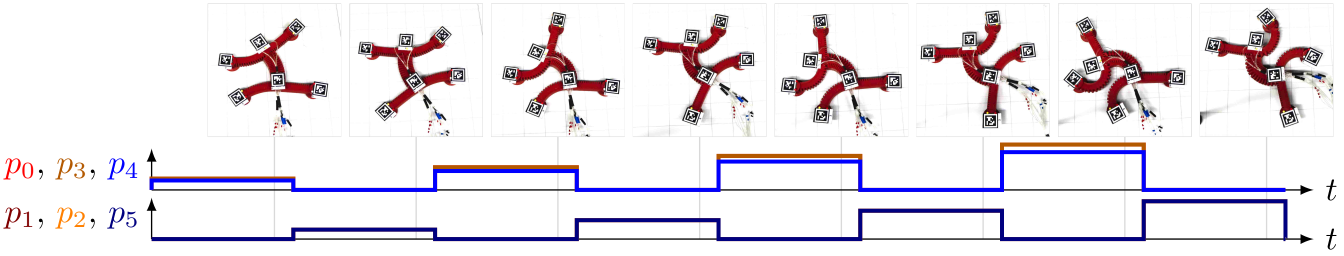

(d) mode 4

Figure 12: Qualitative course of pressure references over time for the four different calibration modes.

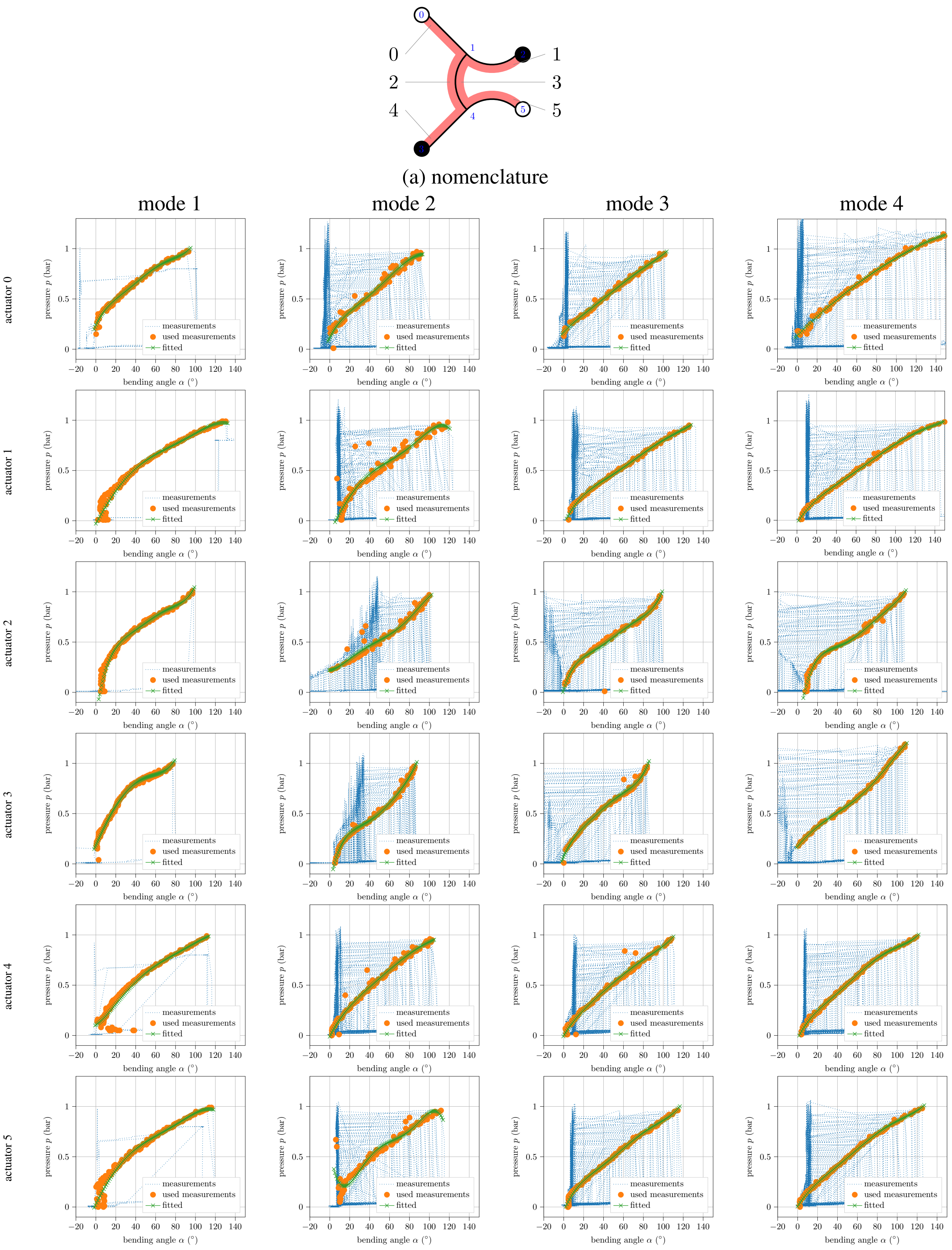

(a) nomenclature

(b) calibration curves

Figure 13: Calibration curves of all limbs of the GeckoBot vS1.1

Table 3: Different calibrations schemes for the bending-pressure relation.

Mode	Description
1	Each actuator is inflated continuously beginning from 0 bar up to 1 bar, while all others remain pressureless.
2	A pressure plateau is applied to a single actuator for 3 seconds; then, it is deflated completely for 2 seconds. In the next round, the level of the pressure plateau is increased by the increment until the plateau reaches 1 bar. This is done for each actuator individually.
3	Same procedure as in mode 2, but here, the same plateau is applied to actuators $(0, 3, 4)$, respectively actuators $(1, 2, 5)$ at the same time.
4	Same procedure as in mode 3, but plateaus for actuators $(0, 3)$ are are starting at 0 bar (like before) and ending at 1.2 bar (instead of 1 bar). Basically the increment for $(0, 3)$ is slightly increased, while the increments for the other actuators remain the same.
